# Role of CD4^+^ T cell mannose binding lectin in *Schistosoma*-induced pulmonary hypertension

**DOI:** 10.1172/jci.insight.202919

**Published:** 2026-07-28

**Authors:** Claudia Mickael, Dara C. Fonseca Balladares, Rahul Kumar, Michael H. Lee, Kevin Nolan, Linda Sanders, Katie J. Tuscan, Ramraj Prasad, Pilar Londono, Fernanda P. Oliveira, Kennedi B. Pyper, Ari B. Molofsky, Rubin M. Tuder, Kurt R. Stenmark, Brian B. Graham

**Affiliations:** 1 Division of Pulmonary Sciences and Critical Care Medicine, Department of Medicine, University of Colorado Anschutz Medical Campus, Aurora, Colorado, USA.; 2 Department of Medicine, UCSF, San Francisco, California, USA.; 3 Lung Biology Center, Zuckerberg San Francisco General Hospital, San Francisco, California, USA.; 4 Department of Translational Immunology, Genentech Inc., South San Francisco, California, USA.; 5 Cardiovascular Pulmonary Research Lab, University of Colorado School of Medicine, Aurora, Colorado, USA.; 6 Division of Respiratory Diseases, Federal University of São Paulo, São Paulo, Brazil.; 7 Department of Laboratory Medicine, UCSF, San Francisco, California, USA.

**Keywords:** Immunology, Pulmonology, Adaptive immunity, Complement, Th2 response

## Abstract

Schistosomiasis is a common cause of pulmonary hypertension (PH) worldwide. It is known that adaptive immunity and specifically CD4^+^ T cells are necessary for experimental disease pathogenesis. The lectin complement system is activated in those infected with schistosomiasis. We tested the hypothesis that lectin complement promotes Th2 CD4^+^ T cell activation, leading to PH in a schistosomiasis exposure model. WT and transgenic mice lacking mannose binding lectin (MBL), and bone marrow chimeras, were experimentally exposed to *Schistosoma mansoni* eggs. PH severity was assessed by hemodynamics and vascular remodeling, and CD4^+^ T cell density and phenotype were assessed by flow cytometry. WT recipients of MBL-knockout bone marrow were protected from *Schistosoma*-induced PH. The protection from PH was associated with fewer Th2 CD4^+^ T cells. In WT mice exposed to *Schistosoma,* CD4^+^ T cell expression of MBL increased. MBL-deficient CD4^+^ T cells had a suppressed Th2 phenotype when exposed to *Schistosoma* antigens. Mice with deficiency of C4, which functions downstream of MBL in the lectin complement pathway, were not protected from *Schistosoma*-induced PH. Mice lacking MBL were not protected from PH caused by hypoxia exposure. MBL in CD4^+^ T cells promotes *Schistosoma*-induced PH.

## Abstract 2

We found an immune cell protein that contributes to lung blood vessel damage due to schistosomiasis, a parasitic infection that causes pulmonary hypertension.

## Introduction

Innate immunity is the first line of defense against pathogens, as it usually precedes adaptive immunity. Innate immunity contributes to adaptive immune activation through mechanisms, including antigen presentation and complement activation. There are 3 proximate complement activation pathways — classical, alternative, and lectin — all leading to the shared formation of complement factor 3 (C3) convertase and downstream activation of the complement cascade.

Schistosomiasis is a common cause of pulmonary hypertension (PH) worldwide. Type 2 immunity is prominent in *Schistosoma*-induced PH, and it is required for the development of experimental disease (1). As a driver of the type 2 inflammatory cascade, IL-4 and IL-13 expression by Th2 CD4^+^ T cells specifically is required for *Schistosoma*-induced PH in mice (2). Complement is activated in humans with schistosomiasis, and the detection of circulating complement fragments was historically used as a test for schistosomiasis (3).

A major function of the complement system is targeting parasites. The parasite tegument and many glycoproteins contain mannose (4), which binds to the host protein mannose-binding lectin (MBL). Higher serum MBL levels and genetic polymorphisms that increase MBL expression are protective against *Schistosoma* infection in humans (5, 6). In contrast, MBL-deficient mice have more eosinophils and immunoglobulin production (7), likely caused by compensatory reactions. Mice with C3 deficiency, which suppresses all 3 complement pathways, have suppressed type 2 inflammation when infected with *Schistosoma*, although they were also protected from liver injury, suggesting the role of complement can be tissue-context dependent (8). In the context of PH, the role of complement activation could be dependent on the etiology; in hypoxia-induced PH, the alternative pathway of complement activation has a causal role (9).

Here, we tested the hypothesis that the complement system acts as a trigger of adaptive immunity, with lectin pathway complement activation being necessary for type 2 inflammation–driven *Schistosoma*-induced PH. Consistent with this hypothesis, we found that mice deficient in MBL in the bone marrow (BM) compartment were relatively protected from PH following experimental *Schistosoma* exposure. We demonstrated a specific requirement for cell-intrinsic MBL in CD4^+^ T cells required to generate a Th2 phenotype. This finding may be broadly applicable to other type 2 inflammation–driven pathologies.

## Results

### MBL deficiency protects mice from Schistosoma-induced PH.

To investigate the functional role of the complement factor MBL in the development of *Schistosoma*-induced PH, we examined mice that lack both isoforms, *Mbl1* and *Mbl2*. These double-knockout mice are hereafter referred to as *Mbl^KO^* mice. WT isogenic mice were used as experimental controls. The mouse model of *Schistosoma*-induced PH used involves sensitizing to *Schistosoma* egg antigen through i.p. injection, followed by challenge with i.v. egg injection. This leads to embolization in the precapillary pulmonary vasculature, while i.p. sensitization is necessary before i.v. challenge for the PH phenotype to develop (10), indicating a requirement for adaptive immunity.

**Figure 1:**
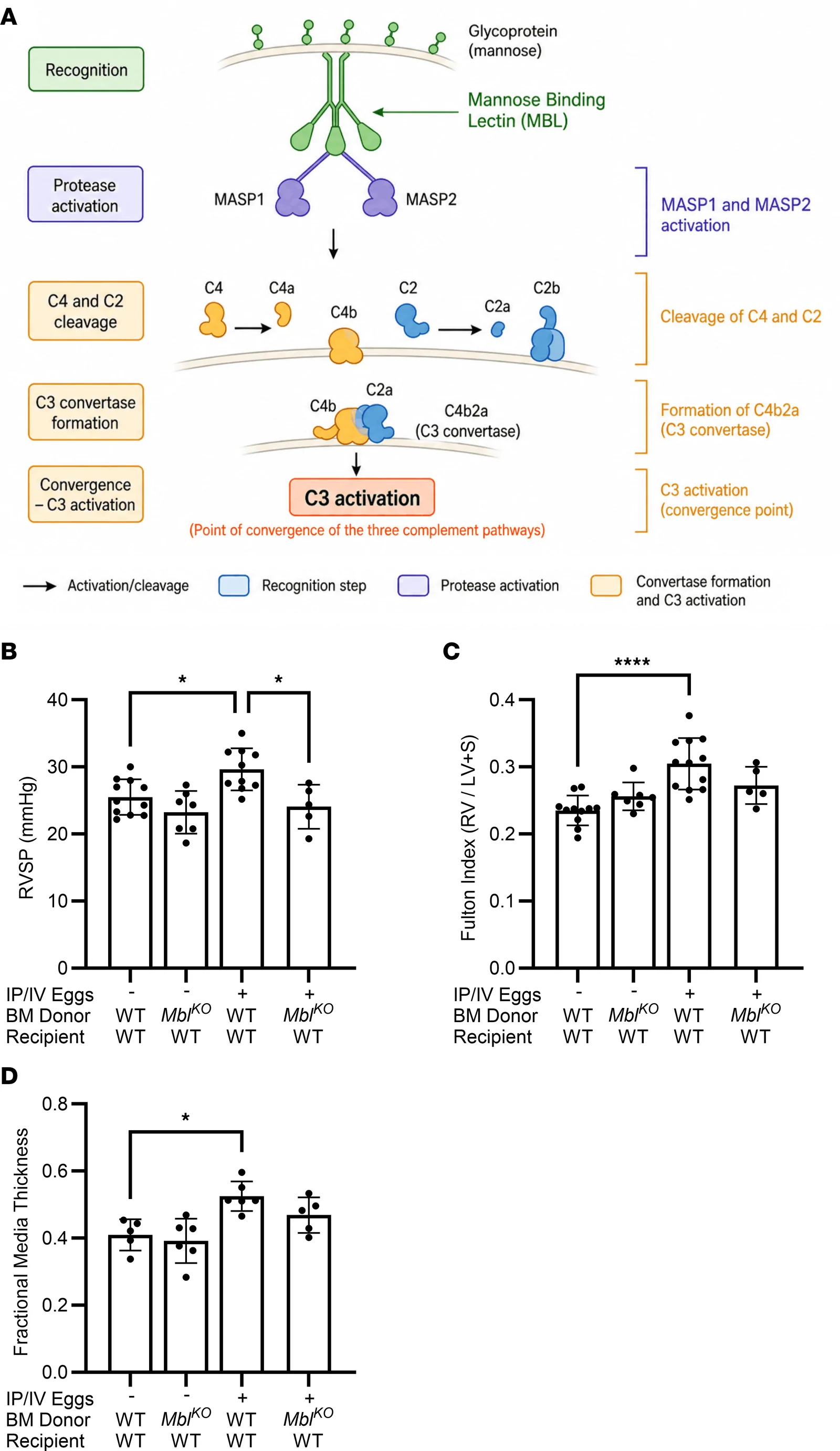
PH phenotype of WT-chimera and *Mbl^KO^*-chimera mice following *Schistosoma* exposure. (**A**) Schematic of the lectin pathway. Mannose on foreign carbohydrates and glycoproteins binds mannose binding lectin (MBL), which forms a complex with mannan-binding lectin-associated serine proteases 1 and 2 (MASP1 and MASP2). This complex enzymatically degrades C4 releasing C4b fragment and C2 releasing C2a fragment. C4b and C2a together form the C4b2a complex, which is functionally a C3 convertase, resulting in C3 activation. C4a and C2b together form the C4a2b complex, which is functionally a C3 convertase, resulting in C3 activation. Active C3 is the point at which the classical and alternative complement pathways converge. This figure was generated by ChatGPT. (**B**) RVSP, (**C**) Fulton index, and (**D**) fractional media thickness in WT recipients of WT or *Mbl^KO^* bone marrow. *N* = 5–12/group; data are shown as the mean ± SD. ANOVA with post hoc Tukey’s test shown, *P < 0.05, **P < 0.01, ***P < 0.001. BM, bone marrow; LV, left ventricle; *Mbl^KO^*, deficiency of both *Mbl1* and *Mbl2*; RV, right ventricle; RVSP, right ventricle systolic pressure; S, septum.

Complement factors including the MBLs are typically described as being expressed by hepatocytes (11, 12), but other organs can also express complement including the BM and BM-derived cells (13). Here, we focused on the immune system and BM-derived complement. We investigated this potential source of MBL by performing BM transplantation (BMT) using *Mbl^KO^* donors and lethally irradiated WT recipients. The recipients, referred to as *Mbl^KO^*-chimeras, were compared with control mice that received WT BM and were treated in a similar manner, denoted as *Mbl^WT^*-chimeras. The *Mbl^WT^*-chimera mice did not exhibit any evidence of a baseline PH phenotype. When the *Mbl^WT^*-chimera mice were subjected to *Schistosoma* egg i.p. sensitization followed by *Schistosoma* egg i.v. challenge, they developed significant PH, as evident by an increase in right ventricle (RV) systolic pressure (RVSP) measured by right heart catheterization, an increase in the RV mass relative to the left ventricle plus septum (Fulton index), and an increase in the thickness of the medial compartment of the pulmonary vasculature, as determined by quantitative analysis of immunostained lung specimens (Figure 1). At baseline, the *Mbl^KO^*-chimera mice also exhibited no discernible pulmonary vascular phenotype. In contrast to the WT-chimeras, following *Schistosoma* egg sensitization and challenge, the *Mbl^KO^*-chimera mice displayed a relative resistance to PH, with no significant increase in RVSP, right ventricular hypertrophy, or medial wall remodeling (Figure 1). These data indicate that the combination of *Mbl1* and *Mbl2*, as expressed by BM-derived cells, is critical for the development of schistosomiasis-induced PH.

### BM MBL deficiency suppresses complement deposition in the lungs of Schistosoma-exposed mice.

Activation of the lectin complement pathway results in C4 and C2 cleavage, the assembly of the C3 and C5 convertases, and fragmentation of C3 and C5. We thus analyzed the lung tissue of the *Schistosoma*-induced PH mice to look for evidence of C3 complement fragment deposition in the lung tissue, using Western blot on whole lung lysates. This revealed in the lung tissue of *Mbl^WT^*-chimeras an increase in the deposition of the terminal C3 degradation product C3d following *Schistosoma* challenge (Supplemental Figure 1; supplemental material available online with this article; https://doi.org/10.1172/jci.insight.202919DS1). In contrast, *Mbl^KO^*-chimera mice challenged with *Schistosoma* had relatively less C3d in the lung tissue. These data suggest that deleting both *Mbl1* and *Mbl2* in the BM compartment suppresses complement activation in the lung tissue.

### BM MBL deficiency reduces CD4^+^ T cell activation.

Given our suspicion that complement may contribute mechanistically to CD4^+^ T cell activation, we analyzed the phenotype of CD4^+^ T cells in the lung tissue of the *Schistosoma*-challenged mice. To exclude intravascular lymphocytes, we administered a fluorescently labeled anti-CD45 antibody i.v. to mark intravascular cells and utilized flow cytometry gating techniques to exclude the marked cells (Supplemental Figure 2A).

**Figure 2:**
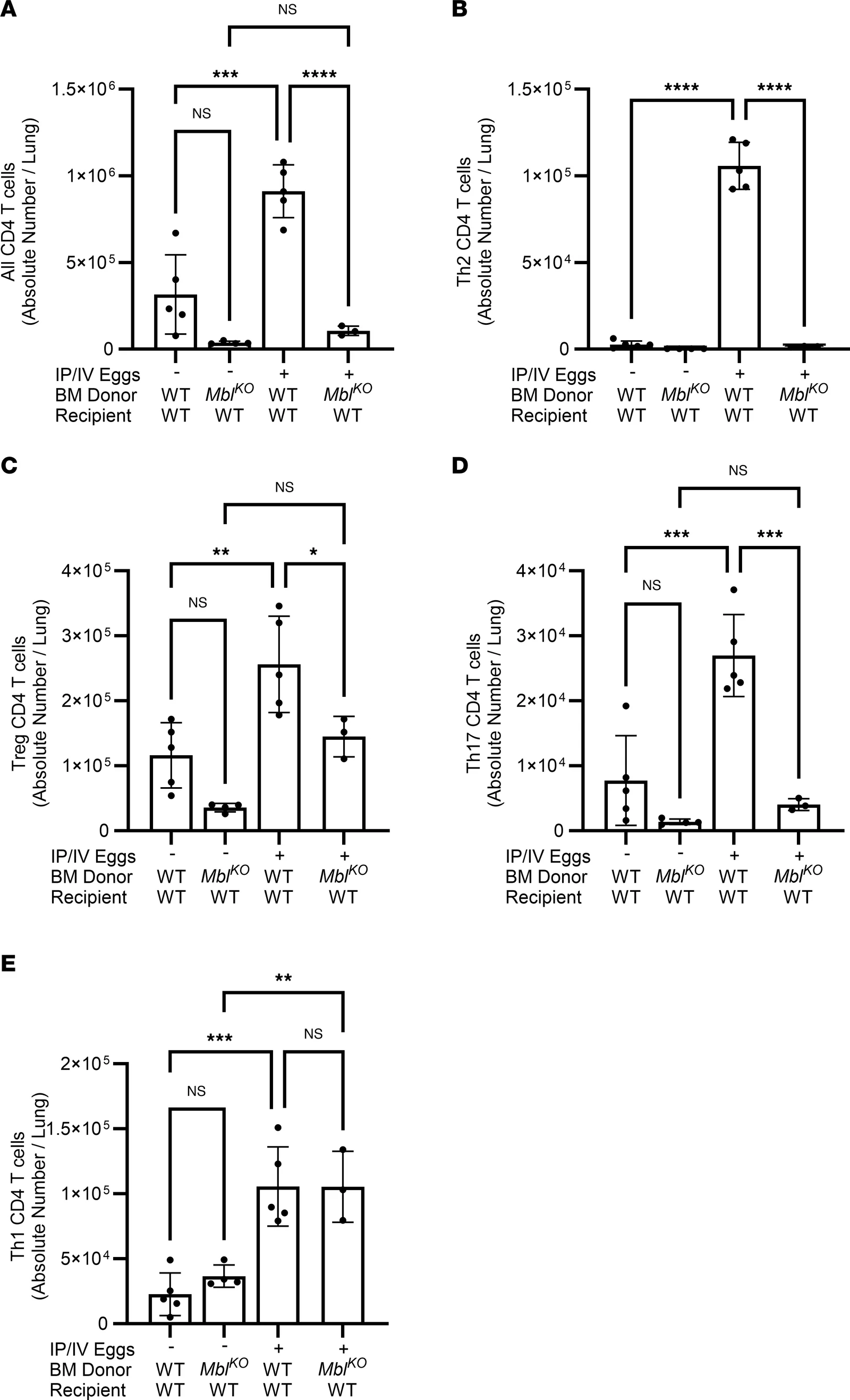
Absolute number of CD4^+^ T cells in WT and *Mbl^KO^*-chimera mice following *Schistosoma* exposure. Absolute number of (**A**) CD4^+^ T cells, (**B**) IL-4^+^ Th2 CD4^+^ T cells, (**C**) IFN-γ^+^ Th1 CD4^+^ T cells, (**D**) IL-17A^+^ Th17 CD4^+^ T cells, and (**E**) FOXP3^+^ Treg CD4^+^ T cells. *N* = 3–5/group; data are shown as the mean ± SD. ANOVA with post hoc Tukey’s test shown, *P < 0.05, **P < 0.01, ***P < 0.001, ****P < 0.0001. BM, bone marrow; *Mbl^KO^*, deficiency of both *Mbl1* and *Mbl2*.

In the *Mbl^WT^*-chimera mice, we observed that *Schistosoma* sensitization and challenge resulted in a 2.9-fold increase in total CD4^+^ T cells and a 39-fold increase in Th2 CD4^+^ T cells in the lungs as compared with that in unchallenged *Mbl^WT^*-chimera mice (Figure 2, A and B). In contrast, there was no significant increase in either total or Th2 CD4^+^ T cells in the *Mbl^KO^*-chimera mice following *Schistosoma* exposure. We also investigated other CD4^+^ T cell phenotypes and found that both Th17 and Treg (Foxp3^+^) CD4^+^ T cells were significantly increased in the *Mbl^WT^*-chimera mice following *Schistosoma* exposure, but this was not seen in the *Mbl^KO^*-chimera mice (Figure 2, C and D). However, both the *Mbl^WT^*-chimera and *Mbl^KO^*-chimera mice displayed a modest increase in Th1 CD4^+^ T cells (Figure 2E), suggesting that lectin complement was upstream of type 2 but not type 1 immunity in *Schistosoma*-exposed mice, although it could also contribute to Th17 and Treg phenotypes as well.

As we had previously observed a requirement for Th2 cells specifically in *Schistosoma*-induced PH (2), we focused on the role of MBL in contributing to type 2 immunity. We conducted IL-4 and IL-13 ELISA assays to evaluate whole lung type 2 inflammation in the mice, finding that the *Schistosoma*-exposed *Mbl^KO^*-chimera exhibited lower levels of IL-4 compared with *Schistosoma*-exposed *Mbl^WT^*-chimera mice (Supplemental Figure 3A). The effect on IL-13 suppression was less pronounced (Supplemental Figure 3B). As an additional measure of type 2 inflammation, we also estimated the volume of peri-egg granulomas using stereology and found that the granuloma volumes were not suppressed in the *Mbl^KO^*-chimera relative to *Mbl^WT^*-chimera mice (Supplemental Figure 3C). These data suggest that the effect of deleting the lectin pathway may relate particularly to the expression of IL-4 by Th2 CD4^+^ T cells.

### MBL expression promotes a Th2 CD4^+^ T cell phenotype by a cell-intrinsic pathway.

**Figure 3:**
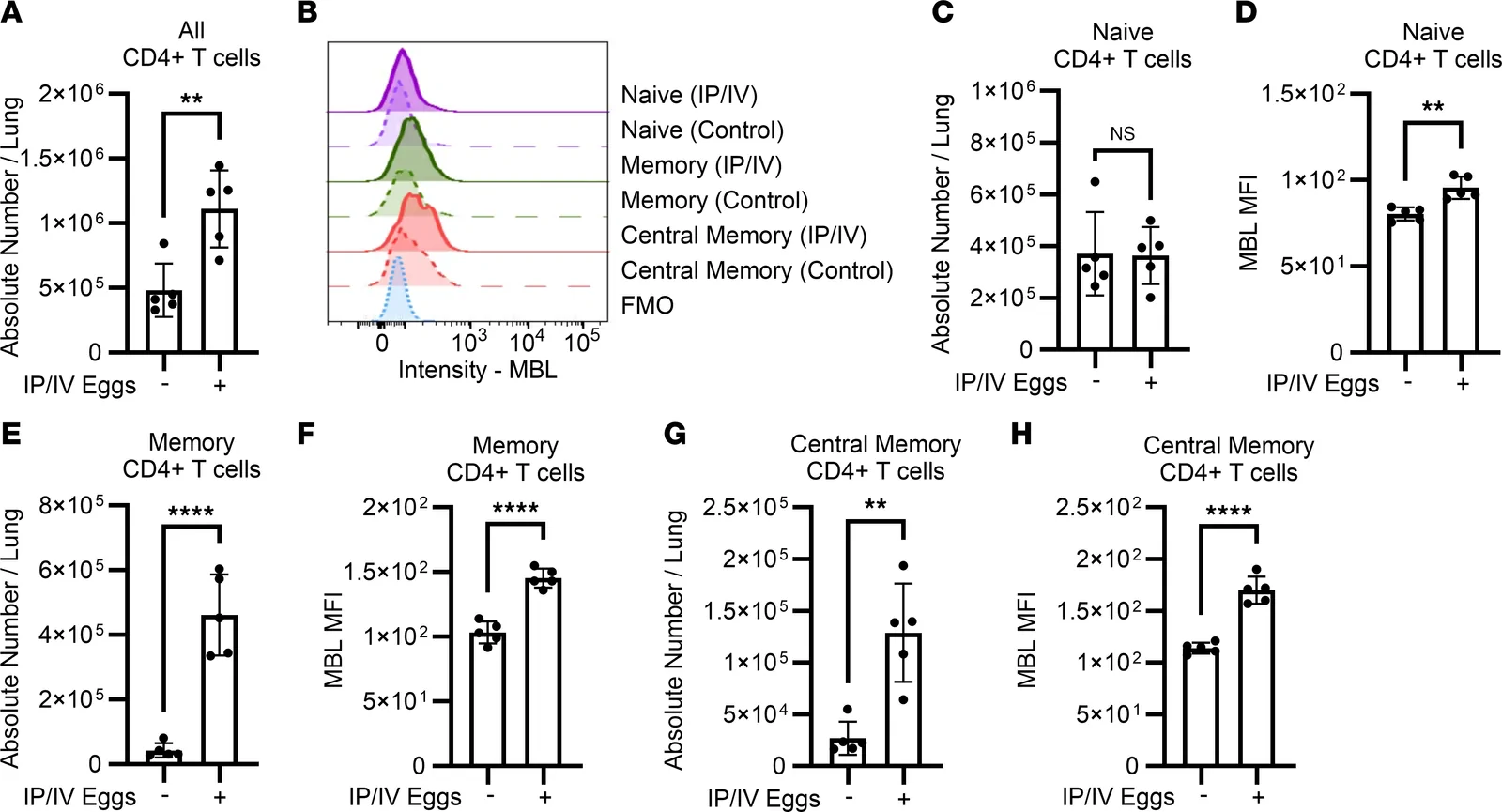
MBL expression by CD4^+^ T cells increases with *Schistosoma* exposure. (**A**) Absolute number of all CD4^+^ T cells. (**B**) Representative expression of MBL in different CD4^+^ T cell populations by flow cytometry, unchallenged or challenged with i.p./i.v. eggs. Absolute number and median fluorescent intensity (MFI) of (**C** and **D**) naive CD4^+^ T cells, (**E** and **F**) memory CD4^+^ T cells, and (**G** and **H**) central memory CD4^+^ T cells. *N* = 5/group; data are shown as the mean ± SD. *t* test, **P < 0.01, ****P < 0.0001.

We then sought to identify which cells within the BM compartment could be necessary to express MBL. We specifically considered the potential guiding role of MBL in the phenotyping of CD4^+^ T cells through an autocrine or cell-intrinsic processes. To test this hypothesis, we first quantified MBL expression by CD4^+^ T cells in *Schistosoma*-exposed mice as compared with control WT mice using flow cytometry (gating strategy in Supplemental Figure 2B). Consistent with this hypothesis, *Schistosoma*-exposed mice had a significant increase in all CD4^+^ T cells, and specifically memory and central memory CD4^+^ T cells, which also expressed higher levels of MBL as compared with control, unexposed mice (as well as naive CD4^+^ T cells, to a more modest extent; Figure 3).

We also queried if Th2 cells are located around the vessels of *Schistosoma*-induced PH mice, which would facilitate pathologic signaling by adjacency. To do so, we used *Il5Cre;Ai14* (IL-5 reporter) mice costained with CD3 to identify Th2 cells. We observed that *Schistosoma*-sensitized and challenged mice had more Th2 cells throughout the entire lung, but in particular many Th2 cells were located immediately adjacent to vessels (Supplemental Figure 4).

We then assessed whether the source of MBL in the CD4^+^ T cells is extrinsic via the extracellular environment versus intrinsic to the CD4 cells. To do so, we first performed a BM chimera experiment, in which a 1:1 ratio of *Mbl^KO^* BM (with the cells marked by CD45.2) was mixed with WT BM (from a CD45.1 donor) and transplanted into a lethally irradiated CD45.1 recipient (Supplemental Figure 5A, and gating strategy in Supplemental Figure 2C). In this manner, we would be able to identify T cells that are MBL expressing (CD45.1) or MBL deficient (CD45.2). We posited that if MBL expression extrinsic to the T cells is necessary, the CD45.1 and CD45.2 T cells would have the same phenotype; whereas if MBL expression intrinsic to the T cells was necessary, then the CD45.1 and CD45.2 T cells would have different phenotypes. Although the sample sizes are very small, we observed trends in fewer CD45.2-marked CD4^+^ T cells, and specifically Th2 CD4^+^ T cells (on average a 9-fold reduction, *P* = 0.08 by paired *t* test), than CD45.1-marked cells (Supplemental Figure 5, B and C). These in vivo data indicated that intrinsic MBL expression by the T cells themselves could contribute to their activation.

**Figure 4:**
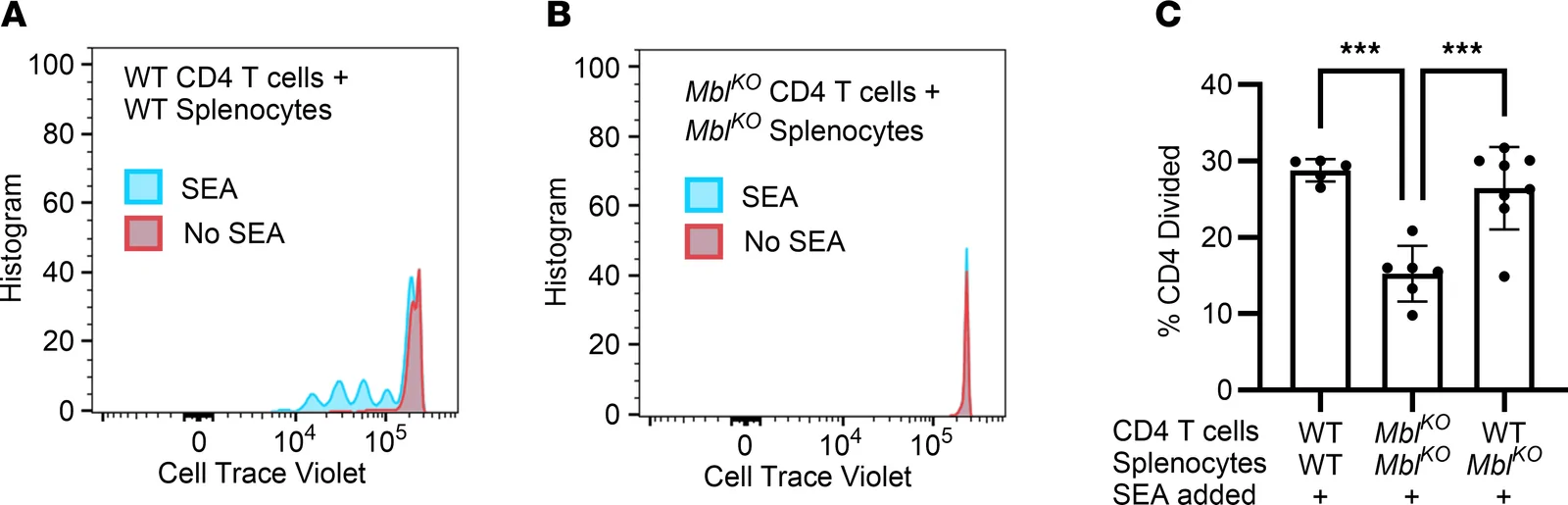
MBL expression by CD4^+^ T cells is required for a Th2 phenotype. (**A**) Histogram of CellTrace Violet expression by WT CD4^+^ T cells, cocultured with WT splenocytes, under conditions of with SEA or no SEA added, demonstrating proliferation with SEA. (**B**) Histogram of CellTrace Violet expression by *Mbl^KO^* CD4^+^ T cells, cocultured with *Mbl^KO^* splenocytes, under conditions of with SEA or no SEA added, demonstrating no T cell proliferation in either condition. (**C**) Quantification of the fraction of CD4^+^ T cells that are divided (have dilution of the CellTrace Violet signal), for WT and *Mbl^KO^* CD4^+^ T cells and splenocytes, all with SEA added, in the combinations shown. *N* = 5–8/group; data are shown as the mean ± SD. ANOVA with post hoc Tukey’s test shown, ***P < 0.001. SEA, soluble egg antigen; *Mbl^KO^*, deficiency of both *Mbl1* and *Mbl2*.

Based on these suggestive in vivo data, we sought to confirm the requirement for MBL to polarize CD4^+^ T cells to a Th2 phenotype using an in vitro approach. We isolated CD4^+^ T cells and splenocytes from the spleens of *Schistosoma*-sensitized WT and *Mbl^KO^* mice. The CD4^+^ T cells were labeled with CellTrace Violet (CTV) ex vivo and then cultured with the splenocytes (serving as antigen-presenting cells) and *S*. *mansoni* soluble egg antigen (SEA) for 96 hours (see summary of experimental design in Supplemental Figure 6A). We read out the phenotype of the CD4^+^ T cells by flow cytometry, quantifying CTV dilution as an indicator of cellular proliferation (Supplemental Figure 6B). We observed that WT CD4^+^ T cells mixed with WT splenocytes underwent significant proliferation (as evidenced by CTV dilution) only after the addition of SEA (Figure 4, A and C). In contrast, *Mbl^KO^* CD4^+^ T cells mixed with *Mbl^KO^* splenocytes and SEA underwent significantly less proliferation (Figure 4, B and C). To confirm that MBL is functionally important in CD4^+^ T cells but not splenocytes, we combined WT CD4^+^ T cells with MBL-deficient splenocytes and observed that the T cell proliferation was comparable to that in cells cocultured with WT splenocytes (Figure 4C).

We considered alternative mechanisms by which T cell activation could be modulated, such as the complement pathway impacting DC function, which would thus indirectly affect the T cell phenotype (14). There are 2 types of conventional DCs, types 1 and 2, referred to as cDC1 and cDC2, respectively. Prototypically, cDC1s activate CD8^+^ T cells, and cDC2s activate CD4^+^ T cells; we recently observed that cDC2s contribute to *Schistosoma*-induced PH (15). By flow cytometry (gating strategy in Supplemental Figure 7), we found that cDC1s but not cDC2s were decreased in the *Mbl^KO^*-chimera mice (Supplemental Figure 8, A and B). We did not observe a change in the number of interstitial macrophages in the *Mbl^KO^*-chimera mice (Supplemental Figure 8C).

### C4-deficient BM does not protect from Schistosoma-induced PH.

**Figure 5:**
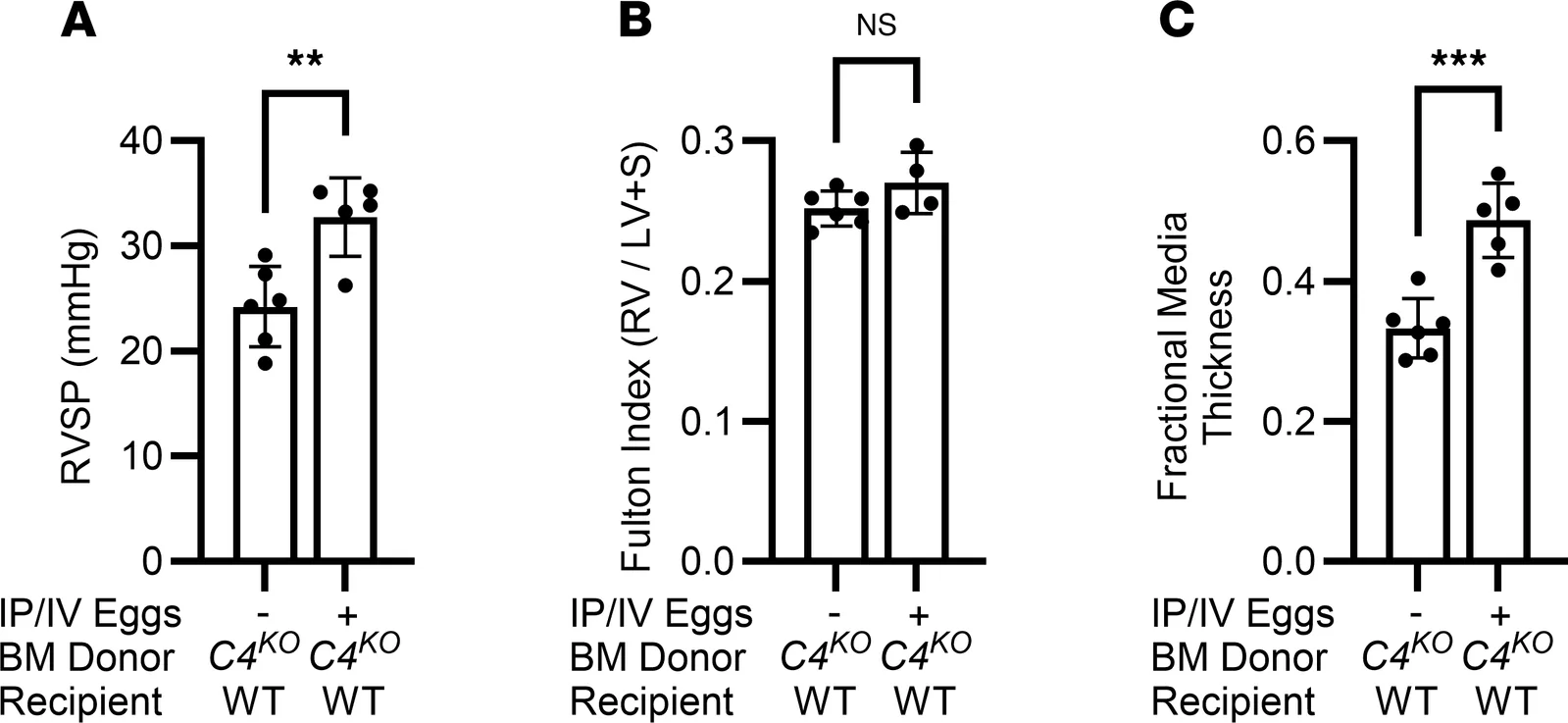
Bone marrow C4 deficiency, a downstream target of MBL, is not protective in *Schistosoma*-induced PH. (**A**) RVSP, (**B**) Fulton index, and (**C**) fractional pulmonary vascular media thickness of *C4^KO^* (C4-deficient) chimera mice, unchallenged or challenged with i.p./i.v. eggs. *N* = 4–6/group; data are shown as the mean ± SD. Unpaired *t* test, **P < 0.01, ***P < 0.001.

Activation of complement factor 4 (C4) is a critical mediator downstream of MBL in the lectin complement pathway: the fragment C4a together with C2b (also activated by the MBL-MASP1-MASP2 complex) forms the C3 convertase that leads to C3 activation converging with the classical and alternative complement pathways. To determine if the canonical extracellular lectin pathway is required for CD4^+^ T cell activation in *Schistosoma*-induced PH, we assessed the phenotype of lethally irradiated WT mice that received C4-knockout (*C4^KO^*) BM, subject to the same i.p. sensitization and i.v. challenge protocol. Interestingly, these mice were not protected from *Schistosoma*-induced PH (Figure 5), suggesting that the extracellular downstream lectin pathway is not required for the function of MBL in activating Th2 cells.

### MBL BM deficiency does not protect from hypoxia-induced PH.

**Figure 6:**
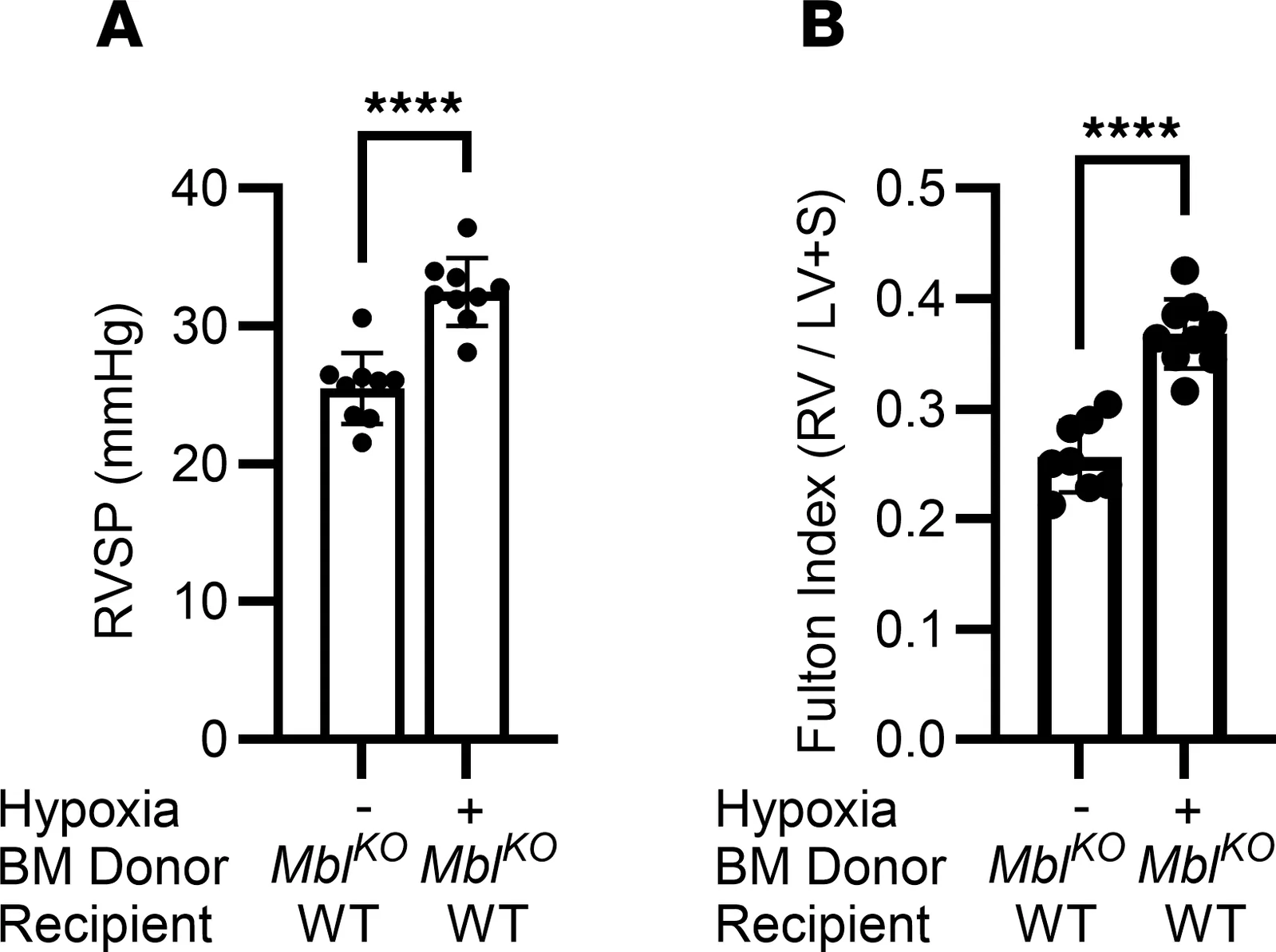
Bone marrow MBL deficiency is not protective in chronic hypoxia-induced PH. (**A**) RVSP and (**B**) Fulton index of WT-chimera and *Mbl^KO^*-chimera mice. *N* = 8–9/group; data are shown as the mean ± SD. Unpaired *t* test, ****P < 0.0001. BM, bone marrow; *Mbl^KO^*, deficiency of both *Mbl1* and *Mbl2*.

As described above, there was reasonable evidence to support the hypothesis that the lectin complement cascade contributes to the pathology present in pulmonary complications of schistosomiasis. We then studied the potential role of the lectin complement pathway in an alternative form of experimental PH, hypoxia-induced disease, which does not have a clear biologic rationale for lectin complement pathway function. We found that the *Mbl^KO^-*chimera mice developed significant PH following 21 days of hypoxia exposure, as evidenced by a significant increase in both RVSP and RV hypertrophy in the hypoxia-challenged mice, as compared with control *Mbl^KO^-*chimera mice maintained in normoxia (Figure 6). These data indicate that MBL in the BM does not contribute to hypoxia-induced PH, which is driven by Th17 immunity (16).

## Discussion

**Figure 7:**
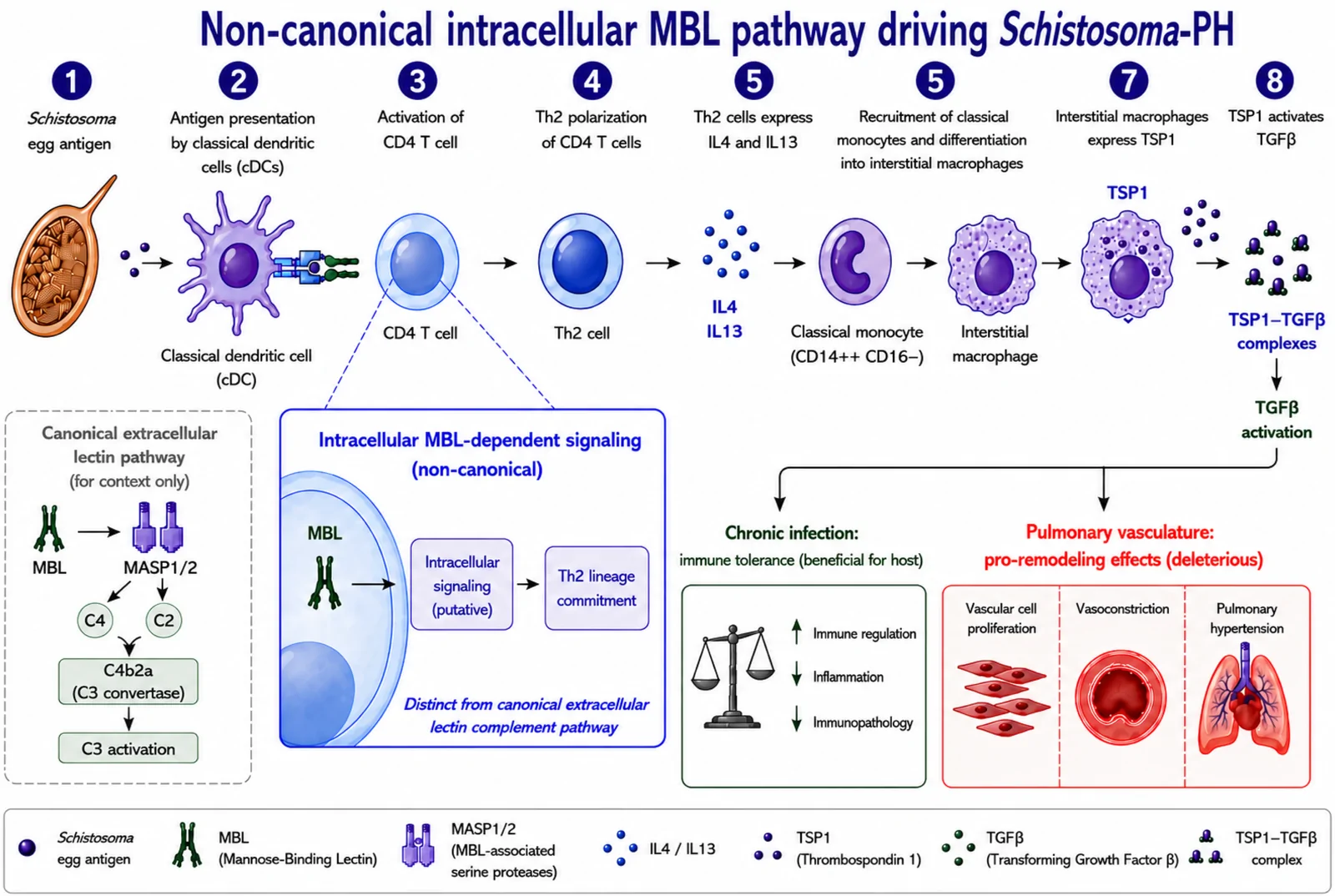
Schematic of the immune cascade promoting *Schistosoma*-induced PH. *Schistosoma* antigens are presented by classical DCs to CD4^+^ T cells, which use MBL to develop a Th2 phenotype. Through the expression of IL-4 and IL-13, the Th2 cells result in the recruitment of classical monocytes, which become interstitial macrophages that express TSP1, causing the activation of TGF-β. In the setting of chronic infection, TGF-β likely promotes a degree of immune tolerance that is beneficial for the host. However, in the context of the pulmonary vasculature, TGF-β causes deleterious proremodeling phenotypes that result in vascular cell proliferation, vasoconstriction, and pulmonary hypertension. Figure generated by ChatGPT.

We found evidence that the lectin complement system is activated in *Schistosoma*-exposed mice and promotes the development of experimental *Schistosoma*-induced PH (Figure 7). MBL proteins expressed by BM-derived cells were required, as deleting both *Mbl1* and *Mbl2* in the BM was found to be protective. We further identified a critical function of MBL in the ability of CD4^+^ T cells to activate to a Th2 phenotype in the context of *Schistosoma* exposure. The requirement for lectin complement pathway activation in the pathogenesis of PH due to *Schistosoma* was dispensable in a noninfectious form of PH following hypoxia exposure, suggesting differential roles of the lectin pathway in different forms of PH. The role of MBL may also be relevant in other Th2-driven pathologies, such as type 2 asthma, or ovalbumin-induced inflammation.

The relevance to human disease is supported by prior observations of more CD4^+^ T cells and evidence of persistent type 2 inflammation in the autopsy lung tissue of individuals who died of *Schistosoma*-induced PH (1, 17). It is possible that targeting type 2 inflammation could benefit those with *Schistosoma*-induced PH, or the initial inflammation could trigger a downstream, self-perpetuating pathology that is now resistant to antiinflammatory treatments. In individuals without concern for active infection, blocking the MBL pathway such as by anti-MBL antibodies could be of benefit (18, 19). However, in individuals who live in an endemic setting and are at risk for reinfection, targeting the lectin complement system would be contraindicated owing to the possibility of compromising the host response to the parasite.

Complement is a critical part of the innate immune system; as such it promotes cell lysis through the formation of the membrane attack complex (MAC) on bacterial membranes, facilitating phagocytosis, promoting inflammation, and helping guide the adaptive immune response. Here, we targeted the lectin pathway, which is specifically activated in response to foreign material by the binding of pattern-recognition plasma molecules such as MBL to carbohydrates or acetylated residues present on organisms such as parasites (20). The bound MBL then activates MBL-associated serine proteases 1 and 2 (MASP-1 and MASP-2), leading to C4 and C2 cleavage. This leads to the assembly of the C3 and C5 convertases, with subsequent cleavage of C3 and C5, which enter the common complement pathway that ends with MAC assembly. Humans have 1 MBL isoform, whereas mice have 2 isoforms; the 2 mouse forms appear to be similar in function (21), with 1 of the 2 (MBL1) lost in the evolution of modern humans (22).

Molecular members of the classical, alternative, and lectin complement pathway have signaling function in expressing cells, independent of activation of MAC. Intracellular C3 and C5, termed the “complosome,” have been described to contribute to the phenotype of Th1 and Th17 CD4^+^ T cells (23–27). C3 and C5 impact T cell phenotypes by regulating metabolism, including through mitochondrial function (23, 25, 28–31). However, a complosome role in Th2 biology has not been previously described to our knowledge. The observation that C4 deficiency in the BM compartment does not protect from *Schistosoma*-induced PH in the same way that MBL deficiency does suggests that the function of MBL does not require downstream C4 activation in this model. Complosome C3 and C5 in Th1 and Th17 cells also do not require the downstream activation of the complement pathway. This is also similar to the observation that DC function requires DC expression of C3AR1 and C5AR1 (32), but C4-deficient DCs do not have suppressed ability to activate T cells (33). Our data that C4 in BM-derived cells appears dispensable for *Schistosoma*-induced PH does not preclude a role for liver-expressed C4 (the majority of C4 is made by the liver) (34, 35), which may contribute to the host response to the parasite.

We observed MBL deficiency also reduced Tregs. In other PH models, Tregs appear to be protective, as their deletion contributes to PH, and reconstituting Treg-deficient animals is protective (36, 37). In the context of *Schistosoma*-induced PH, Th2 cells mechanistically contribute to pulmonary vascular pathology without being affected by levels or activation of Tregs (2), as compared with the protective role of Tregs in other forms of PH.

There are several potential mechanisms by which MBL expression in CD4^+^ T cells could be increased. MBL is an acute phase protein, with increased expression in settings of inflammation, such as ischemia-reperfusion and oxidative stress (38), as well as infection. Hypoxic pathway mechanisms are another candidate driver (39). In CD4^+^ T cells, the HIF2α isoform has been linked to a Th2 phenotype (40).

Complement links innate and adaptive immunity. We have found Th2 CD4^+^ T cells are required for *Schistosoma*-induced PH (1, 2). C3aR, C5aR, and CD46 (a regulator of complement activation) are all expressed by T cells (41), and all act as costimulatory signals for T cells (32, 42). C3aR is expressed in the lysosomes of naive T cells and transported to the cell membrane of activated T cells (43). We observed the function of CD4^+^ T cells is controlled by intrinsic MBL expression, likely acting at differentiation stages prior to their full activation, as MBL expression was lower in activated cells. There is evidence in other settings of an autocrine function for immune cell-derived complement, as T cells from C3-deficient patients cannot generate IFN-γ even in C3-sufficient serum (43). *C3ar1^–/–^* and *C5ar1^–/–^* mice have defective T cell activation (32, 44) and shift their phenotype to Tregs (45). Tregs express C3aR and C5aR, and signaling through these receptors decreases Foxp3 expression and inhibits Treg function (45). We found that MBL-deficient chimeras had fewer cDC1s, although this does not explain the protection observed, as experimentally depleting cDC1s using *Batf3^–/–^* mice resulted in more severe *S*. *mansoni*–induced PH (15). We previously observed that cDC2s in particular contribute to *Schistosoma*-induced PH (15); here, we found that the MBL depletion in CD4^+^ T cells did not impact cDC2 density, consistent with the role of cDC2s being proximate to T cell activation.

There are broader implications related to the relevance of MBL in schistosomiasis infection. Genetic polymorphisms in the MBL promoter that lead to decreased expression and lower plasma MBL are associated with increased schistosomiasis infection (6). MBL genetic polymorphisms and higher plasma levels are also associated with more severe liver disease (46, 47), which likely contributes to the development of clinical PH by promoting egg embolization via portocaval shunts (48).

A limitation in our work is that we did not precisely knockout MBL in CD4^+^ T cells in vivo. We are not aware of these mice being developed and would be challenged by needing to target both MBL isoforms in a cell-specific manner. We were able to isolate the requirement to the BM compartment and then focused specifically on the role of MBL in CD4^+^ T cells using our in vitro system.

In the BM transplant experiments, we used mixtures of male and female donor cells transplanted into only female recipients. Sex-mismatch status between donor and recipient can independently stimulate the immune system and is a risk factor for graft-versus-host disease (49, 50); thus, some of the immune pathways stimulated and modulated by MBL could be independent of *Schistosoma*-driven immunity. However, the greatest signal reported is for female donors into male recipients, potentially related to increased immune memory from prior pregnancies in the donor and/or Y chromosome–derived antigens in the recipient (49, 50). None of the donor or recipient mice we used had been previously pregnant: all were dedicated for experimental purposes.

Another limitation is the uncertainty of why whole-lung IL-4 and IL-13 concentration did not correlate as strongly with CD4^+^ Th2 density and the PH phenotype. This may be due to other cells expressing IL-4 and IL-13, which could include ILC2s, macrophages, mast cells, eosinophils, and basophils. This observation may also relate to location of cytokine expression, either concentrated in the pulmonary vasculature or that in other lung compartments, possibly in relation to peri-egg granulomas. Creating lung lysates will result in average cytokine concentrations across the entire tissue, leading to an apparent discrepancy as compared to assessing perivascular cells specifically.

In conclusion, we found that lectin complement pathway MBL proteins contribute to the pathogenesis of experimental schistosomiasis-induced PH by mechanistically contributing to the activation of CD4^+^ T cells to a Th2 phenotype. Therapeutically targeting this pathway to prevent or treat clinical *Schistosoma*-induced PH specifically should be considered with caution, as this pathway’s contribution is likely at the earliest stages of disease development, and host immunity should ideally be preserved in the event of ongoing infection or reinfection with the parasite. However, this may be a promising therapeutic target in other Th2-driven diseases such as asthma.

## Methods

### Sex as a biological variable

In the BM chimera experiments, the donors were males and females, with the BM typically mixed in a 1:1 ratio between sexes, and the recipients were only female animals. The animals that were the source of CD4^+^ T cells and splenocytes for in vitro experiments were both male and female mice, as were the C4-knockout mice. For the experiments including both female and male animals, no difference was observed between sexes. The BM recipients were female as PH is more prevalent in females (51).

### Animals

WT (C57BL6/J, strain 000664), *Mbl1^–/–^Mbl2^–/–^* (strain 006122, referred to as *Mbl^KO^* throughout), and *C4^–/–^* (strain 003643, referred to as *C4^KO^*) mice were purchased from The Jackson Laboratories. These mice were housed at the University of Colorado Anschutz Medical Campus (CUAMC) specific pathogen–free animal facilities. These animal experiments were conducted in accordance with protocol 000057 approved by the CUAMC Institutional Animal Care and Use Committee. Additionally, *Il5-Cre/^+^;Rosa26(CAG-tdTomato)/^+^* (termed IL-5 reporter) mice were provided by UCSF. These mice were housed in pathogen-free animal facilities at UCSF, and the UCSF animal experiments were conducted in accordance with protocol AN208579-00C, approved by the UCSF Institutional Animal Care and Use Committee.

### Experimental treatments

#### BMT.

BM cells were harvested from the femurs of 5- to 6-week-old donor mice. Recipient mice underwent lethal irradiation (MultiRAD350 X-Ray Irradiator, Precision X-Ray Irradiation) at a dose of 4.5 Gy administered twice at a 4-hour interval. BMT was performed immediately after the second irradiation dose, with mice receiving 4 × 10^6^ to 6 × 10^6^ BM cells via retro-orbital injection. To prevent infection, animals were provided a sulfonamide-enriched Uniprim diet (Teklad Global) for 3 weeks. Experimental procedures were initiated 4 weeks after BMT to allow for hematopoietic chimerism.

#### Schistosoma mansoni exposure.

Mice were exposed to *S*. *mansoni* following established protocols (52, 53). Briefly, mice were sensitized i.p. with *Schistosoma* eggs and subsequently challenged i.v. with eggs. Unexposed mice served as controls. For catheterization studies, mice underwent right heart catheterization 7 days after i.v. challenge. For flow cytometry studies, mice were sacrificed 3 days after i.v. challenge, timed to coincide with peak inflammation.

#### Hypoxia challenge.

For hypoxia experiments, mice were placed in a controlled chamber and exposed to hypobaric hypoxia in a chamber simulating 5,000 m altitude (pressure ≈428 Torr), equipped with automated O_2_ control and monitoring (BioSpherix ProOx controller), as previously described (54).

### PH experimental endpoints

RVSP and RV hypertrophy were assessed as previously described (52, 55). Briefly, mice were sedated with ketamine/xylazine and transtracheally ventilated. The abdominal and thoracic cavities were opened, and a 1Fr pressure-volume catheter (PVR-1035, Millar Instruments) was placed through the RV free wall to transduce the RV pressure. The Fulton index was assessed by resecting the RV free wall from the left ventricle and septum, weighing them separately, and calculating the ratio. The lung tissue was then harvested and snap-frozen for subsequent protein measurement or inflated in agarose for FFPE. Pulmonary vascular remodeling was assessed by immunostaining FFPE tissue for α-smooth muscle actin (αSMA; Invitrogen 14-9760-82) using a published protocol (56). Images were captured with a Nikon Eclipse 80i microscope, and the vascular media were identified using image processing software (Image-Pro 10, Media Cybernetics). The average radii of the outer and internal perimeters of the medial layer were quantified, and the fractional medial thickness was calculated as the difference in these radii divided by the external media radius. The optical rotator stereology method (57) was used to estimate peri-egg granuloma volumes surrounding a single egg on images from hematoxylin and eosin–stained slides.

### In vitro cell experiments

CD4^+^ T cells were isolated from splenocytes harvested from WT and *Mbl^–/–^* mice 15 days after i.p. sensitization with *S*. *mansoni* eggs. Splenocytes were processed using a CD4^+^ Cell Isolation Kit (Miltenyi Biotec) and labeled with CTV according to the manufacturer’s instructions. Labeled CD4^+^ T cells were mixed with CD4-deficient splenocytes at a 1:5 ratio in 96-well U-shaped–bottom plates. Cultures were stimulated with SEA (50 μg/mL) prepared from *S*. *mansoni* eggs using published protocols (58). Cells were cultured in RPMI 1640 supplemented with 2 mM L-glutamine, 50 μM 2-mercaptoethanol, 10% fetal calf serum, and penicillin/streptomycin at 37°C in 5% CO_2_. Cultures were incubated for 72 hours, and proliferation was assessed by flow cytometry based on CTV dilution. After 72 hours of culture, cells were harvested, washed with PBS, and stained with a viability dye to exclude dead cells. Samples were acquired using a BD LSR Fortessa flow cytometer (BD Biosciences) with appropriate compensation controls. Proliferation was assessed by dilution of CTV within the CD4^+^ T cell population. Data were analyzed using FlowJo software (version 10.10.0).

### Protein quantification

#### Tissue collection and protein extraction.

Lung tissues were harvested from WT and *Mbl^–/–^* chimera mice following *S*. *mansoni* exposure. Tissues were rinsed in cold PBS, snap-frozen, and homogenized in RIPA buffer supplemented with protease inhibitor cocktail. Homogenates were incubated on ice for 20 minutes and then centrifuged at 12,000*g* for 15 minutes at 4°C to remove debris. Supernatants were collected, and protein concentration was determined using the Bio-Rad protein DC assay.

#### Western blot analysis.

Equal amounts of protein (20 μg per lane) were mixed with Laemmli sample buffer and boiled at 95°C for 5 minutes. Samples were resolved on 4%–10% SDS-PAGE gradient gels and transferred to PVDF membranes using a wet transfer system at 100 V for 30 minutes. Membranes were blocked in Superblock in TBST for 1 hour at room temperature. Blots were incubated overnight at 4°C with an anti-C3 primary antibody (Supplemental Table 1). After washing, membranes were incubated with HRP-conjugated secondary antibody for 1 hour at room temperature. Protein bands were visualized using chemiluminescent substrate, ECL, and imaged with Bio-Rad ChemiDoc. Band intensities for C3 and its fragments were quantified using ImageLab software and normalized to β-actin.

#### IL-4 and IL-13 protein quantification in tissue lysates.

Commercial ELISA kits were used for the quantification of IL-4 and IL-13 (DuoSet kits DY404 and DY413, respectively; R&D Systems) using whole-lung lysates from unexposed and *Schistosoma*-exposed mice, following the manufacturer’s instructions.

### Flow cytometry

#### Lung tissue digestion and cell isolation.

Lungs were harvested from WT and *Mbl^–/–^* chimera mice following Schistosoma mansoni exposure. Tissues were perfused with PBS, minced, and digested in Liberase (Roche) (0.4 mg/mL) and in RPMI 1640 at 37°C for 30 minutes. Digested tissue was passed 5 times through a 16-gauge needle, followed by 5 times through an 18-gauge needle, and then through a 100 μm cell strainer to obtain single-cell suspensions. Red blood cells were lysed using ACK lysis buffer, and the cells were washed and resuspended in complete RPMI medium.

#### Cell stimulation.

Cells were cultured in RPMI 1640 supplemented with 10% FBS, 2 mM L-glutamine, 50 μM 2-mercaptoethanol, and penicillin/streptomycin. Single-cell suspensions were plated in 6-well plates and stimulated in vitro with a 500X cell stimulation cocktail of phorbol 12-myristate 13-acetate, ionomycin, brefeldin A and monensin (Invitrogen) for intracellular cytokine accumulation. Cultures were maintained for 6 hours at 37°C in 5% CO_2_.

#### Flow cytometry and absolute cell counting.

After stimulation, viability dye was added to exclude dead cells. Then, cells were stained with fluorophore-conjugated antibodies (Supplemental Table 2) targeting surface markers, including CD45, CD3, and CD4. For intracellular cytokine determination, cells were fixed, permeabilized, and stained with the intracellular antibodies IL-4, IFN-γ, and IL-17A using the Intracellular Fixation and Permeabilization buffer set (eBioscience). For nuclear cytokine determination, Foxp3/Transcription Factor Staining Buffer Kit (Tonbo Biosciences) was used according to the kit instructions. Absolute numbers of CD4^+^ T cell subsets were determined using counting beads (AccuCount, Spherotech) according to the manufacturer’s instructions. Samples were acquired on a BD LSRFortess flow cytometer, and data were analyzed using FlowJo software. Gating strategy included live CD45^+^ lymphocytes to CD4^+^ T cells, and absolute counts were calculated based on bead recovery.

### Protein detection in IL-5 reporter mice

The *Il5Cre;Ai14* (IL-5 reporter) mice underwent *Schistosoma* egg sensitization and challenge as described above, and the tissue was collected at the time points described. Following CO_2_ euthanasia, the mice were transcardially perfused with 10 mL of DPBS, and the lung lobes were dissected and stored in 4% PFA for 1–3 days at 4°C followed by cryoprotection (30% sucrose). The lung lobes were subsequently frozen in OCT (Thermo Scientific) on dry ice and stored at –80°C. Slide-mounted 10 μm thin sections were collected on a cryostat (Leica), thawed, washed (with Dulbecco’s PBS [DPBS]), and blocked (1 hour, DPBS/0.4% Triton X-100/5%, goat serum). Samples were then incubated in primary antibodies (rabbit anti-dsRed (Takara 632496, 1:300), Syrian hamster-anti-CD3ε (500A2, BD Biosciences, 553238, 1:300) diluted in blocking solution overnight at 4°C. Samples were washed (DPBS/0.05% Triton X-100, 5 minutes, 3 times) and incubated in secondary (1:1,000, donkey anti-rabbit IgG AF555; Thermo Scientific, A31572), goat anti-hamster IgG AF647 (Thermo Scientific, A21451), or conjugated (anti-aSMA AF405; R&D Systems, IC1420V025, 1:300)) antibodies in blocking solution at room temperature for 1 hour. Samples were washed as previously described, mounted in DAPI Fluoromount-G (Thermo Scientific), and imaged. Confocal images were acquired using a Leica SP8 laser scanning confocal equipped with an AOBS tunable detection pathway, a white light laser that excites between 470 and 670 nanometers, and a lighting deconvolution module. An Apo IMM (H2O Dipping) 20× 1.95 mm working distance objective was used. Z steps were acquired every 8 μm. *Z*-stacks were rendered in 3D and quantitatively analyzed using Bitplane Imaris v9.8 software package (Oxford Instruments). Individual cells were annotated using the Imaris surface function based on fluorescent reporter signal and size/morphological characteristics, with background signal in unrelated channels excluded. 3D reconstructions of vessels and airways were generated using the Imaris surface function based on the fluorescent αSMA signal.

### Figure generation

We used generative AI (ChatGPT, version 5) to generate Figures 1 and 7 from April 27, 2026, to August 2026. A rough draft of a schematic pathway was uploaded to ChatGPT, with prompts including, “Can you help me redraw this figure in a clearer way, making more representative drawings?”

### Statistics

All results are reported as mean ± SD. Differences between 2 groups were assessed by paired or unpaired 2-tailed *t* test; for ≥ 3 groups, differences were assessed by 1-way ANOVA followed by post hoc Tukey’s test. *P* values of less than 0.05 were considered statistically significant. Prism (v10.1, GraphPad) was used for statistical analysis and graphing.

### Study approval

The present studies in animals were reviewed and approved by CUAMC Institutional Animal Care and Use Committee and UCSF Institutional Animal Care and Use Committee.

### Data availability

Data are available in the Supporting Data Values file and from the corresponding authors upon reasonable request.

### Authorship contributions

CM: conceptualization, data curation, formal analysis, funding acquisition, investigation, methodology, visualization, and writing of the original draft. DCFB: data curation, formal analysis, investigation, visualization, and review and editing of the manuscript. RK: data curation, formal analysis, investigation, methodology, visualization, and review and editing of the manuscript. MHL: investigation, methodology, and review and editing of the manuscript. KN: investigation and review and editing of the manuscript. LS: investigation and review and editing of the manuscript. KJT: data curation, investigation, and review and editing of the manuscript. RP: investigation, methodology, and review and editing of the manuscript. PL: investigation and review and editing of the manuscript. FPO: formal analysis, investigation, visualization, and review and editing of the manuscript. KBP: data curation, investigation, visualization, and review and editing of the manuscript. ABM: formal analysis, methodology, visualization, and review and editing of the manuscript. RMT: conceptualization, methodology, and review and editing of the manuscript. KRS: conceptualization, funding acquisition, methodology, project administration, and review and editing of the manuscript. BBG: conceptualization, formal analysis, funding acquisition, methodology, project administration, writing of the original draft, and review and editing of the manuscript.

## Conflict of interest

BBG has consulted for Merck.

## Funding support

This work is the result of NIH funding, in whole or in part, and is subject to the NIH Public Access Policy. Through acceptance of this federal funding, the NIH has been given a right to make the work publicly available in PubMed Central.

- NIH grants R01HL135872 (to BBG), P01HL152961 (to RMT, KRS, and BBG), K08HL168310 (to MHL), K01HL161024 (to CM), and R01AI162806 (to ABM).
- US Department of Defense grant W81XWH2210457 (to MHL).
- American Heart Association grant 19CDA34730030 (to RK).
- ATS Early Career Investigator Award in Pulmonary Vascular Disease Program (to RK).
- NIH/National Institute of Allergy and Infectious Diseases contract HHSN272201700014I to the Schistosomiasis Resource Center of the Biomedical Research Institute.

## Supplementary Material

Supplemental data

Unedited blot and gel images

Supporting data values

## Version 1 07/28/2026

In-Press Preview

## Version 2 09/08/2026

Electronic publication
