# Supplementary material for: Role of CD4+ T cell mannose binding lectin in Schistosoma-induced pulmonary hypertension
Source: JCI Insight. 2026 Jul 28;11(17):e202919. doi: 10.1172/jci.insight.202919 (PMC13564070; doi:10.1172/jci.insight.202919)
Supplement: Supplementary file 1 [file jciinsight-11-202919-s193.pdf]

Online Supplement

CD4 T Cell Mannose Binding Lectin is Required for *Schistosoma*-Induced Pulmonary Hypertension

Mickael et al.

Supplemental Table 1. Antibodies used for western blot.

Antibody Specificity	Format	Final Conc.	Manufacturer	Cat. Number
Rabbit Anti-b-actin	Polyclonal	1/5000	Cell Signaling Technology	4967
Rabbit Anti-C3	Polyclonal	1/3000	Abcam	A16781
Goat anti-Rabbit	IgG H+L	1/5000	Vector	PI-1000

Supplemental Table 2. Antibodies used for murine flow cytometry.

Antibody Specificity	Fluorochrome	Clone	Final Conc.	Manufacturer
Anti-CD3	APC/C7	17A2	1/100	Tonbobiosciences
Anti-CD3	eFluor™450	17A2	1/100	Invitrogen
Anti-CD3	FITC	17A2	1/100	BD Pharmingen™
Anti-CD4	BV510	RM4-5	1/100	BioLegend
Anti-CD4	APC	RM4-5	1/100	BD Pharmingen™
Anti-IL4	Alexa™Fluor 488	11B11	1/100	BDBiosciences
Anti-IL17A	APC	eBio1787	1/100	Invitrogen
Anti-IFNγ	PE	XMG1.2	1/100	Invitrogen
Anti-FoxP3	PE	MF23	1/100	BD Pharmingen™
Anti-IV CD45	PerCp	104	1/100	BioLegend
Anti-CD45	BV650	104	1/100	BioLegend
Anti-CD45	BV785	30-F11	1/100	BioLegend
Anti-IA/IB	APC/Cy7	M5/114.15.2	1/100	BioLegend
Anti-B220	eFluor™450	RA-3682	1/100	Invitrogen
Anti-Ly6G	eFluor™450	1AB-Ly6g	1/100	Invitrogen
Anti-SIGLECF	PE-CF594	E50-2440	1/100	BD Horizon
Anti-CD11b	BV510	M1/70	1/100	BD Horizon
Anti-CD11c	PE/Cy7	N418	1/100	Invitrogen
Anti-CD64	APC	X54-5/7.1	1/100	BioLegend
Anti-CD103	BV605	2E7	1/100	BioLegend
Anti-CD301b	PE	URA-1	1/100	BioLegend
Anti-CD24	FITC	M1/69	1/100	BioLegend
Anti-CD8b.2	PE-Cy7	53-5.8	1/100	BioLegend
Anti-CD62L	BV650	MEL-14	1/100	BioLegend
Anti-CD44	PE	IM7	1/100	BioLegend
Anti-IL33R	PE	RMST2-2	1/100	eBiosciences
Anti-CD45.1	BV785	A20	1/100	BioLegend
Anti-CD88	BV711	20/70	1/100	eBiosciences
Anti-MBL	FITC	Polyclonal	1/100	Mybiosource.com/ MBS2055689
Viability dye	e450	N/A	1/1000	Invitrogen
Viability dye	Near IR	N/A	1/1000	Invitrogen

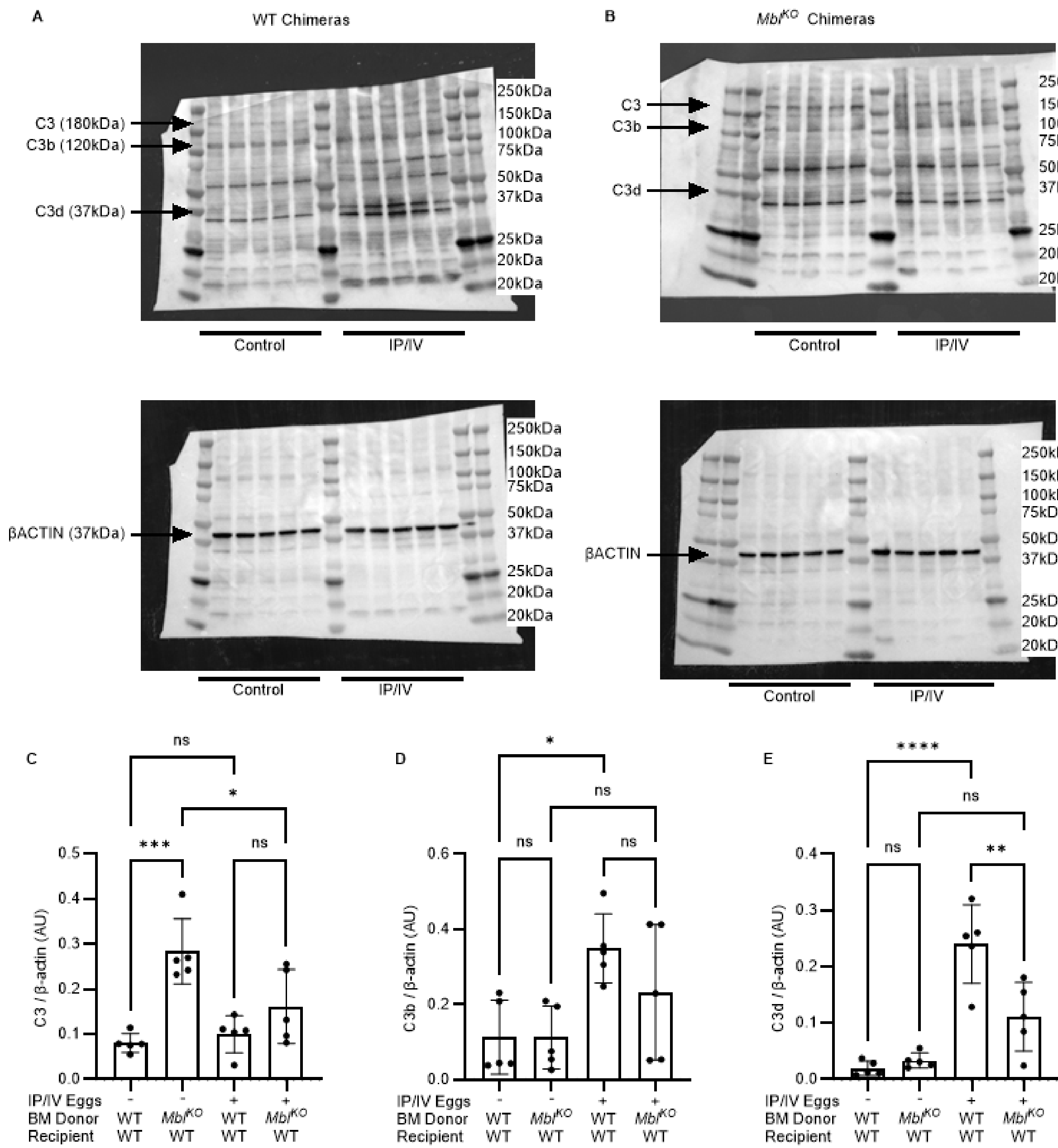

Supplemental Figure 1. Complement component C3 protein and fragments in the lungs of wildtype and *Mbl*$^{-/-}$ chimera mice following *Schistosoma* exposure. Western blots of (A) wildtype control and IP/IV mice, and (B) *Mbl*$^{-/-}$ chimera control and IP/IV mice. Densitometry analysis of (C) intact C3, (D) fragment C3b, and (E) fragment C3d, normalized for βACTIN. Mean +/- SD plotted; ANOVA with post-hoc Tukey test shown. Abbreviations: BM: bone marrow; IP: intraperitoneal; IV: intravenous; *MBL*$^{-/-}$: mannose binding lectin 1 and 2 knockout; WT: wildtype. P values: ns: non-significant; *P<0.05, **P<0.01, ***P<0.001, ****P<0.0001.

Supplemental Figure 2. Flow cytometry gating strategies for T cells.

A: Representative gating for T cells. (i) Minimum size requirement. (ii) and (iii) Selection for single cells. (iv) Positive for CD45, and negative for the viability dye. (v) CD3+. (vi) CD4+. (vii) Distinguishing naïve from memory based on CD62L and CD44 expression.

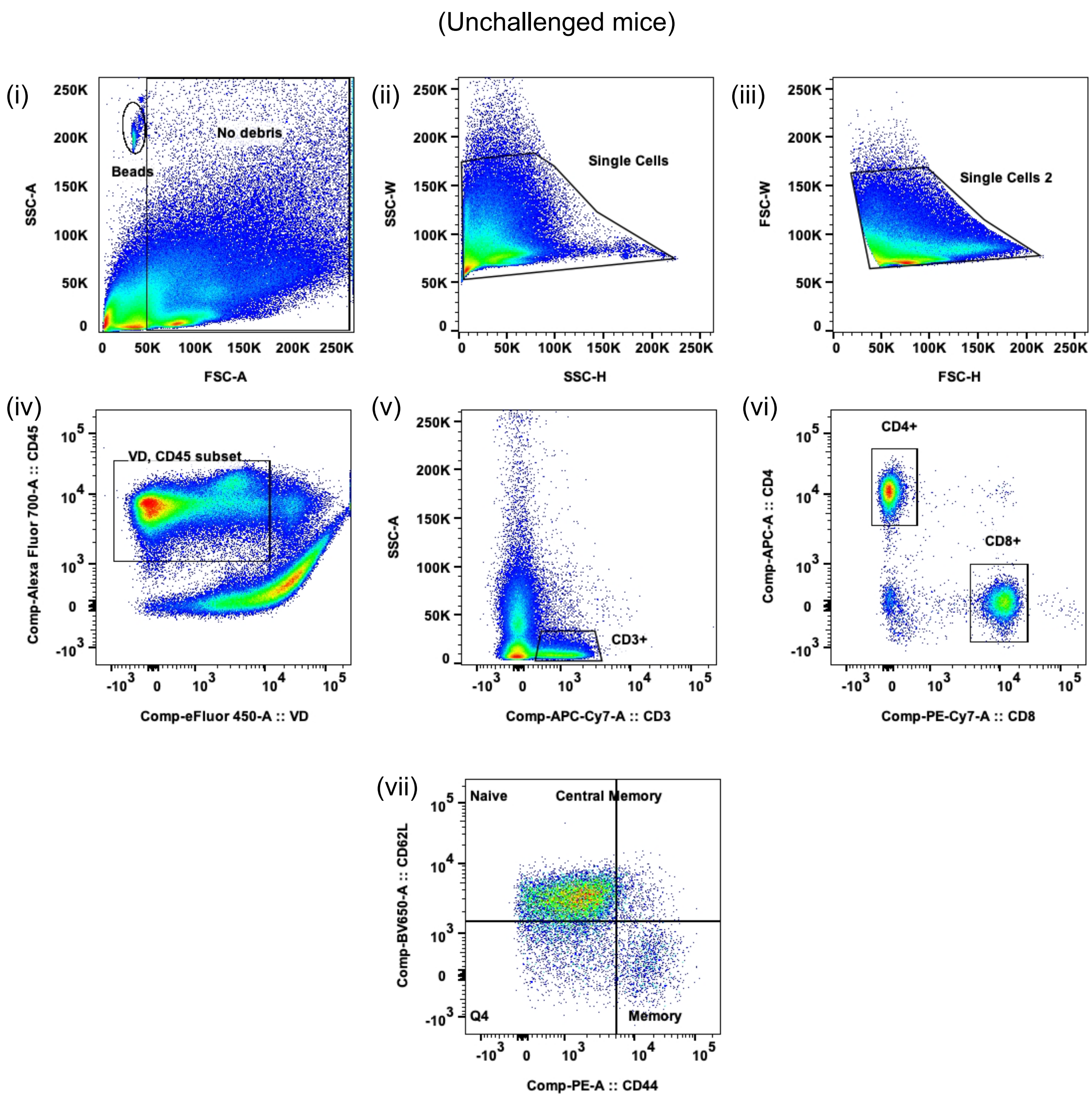

(IP/IV challenged mice)

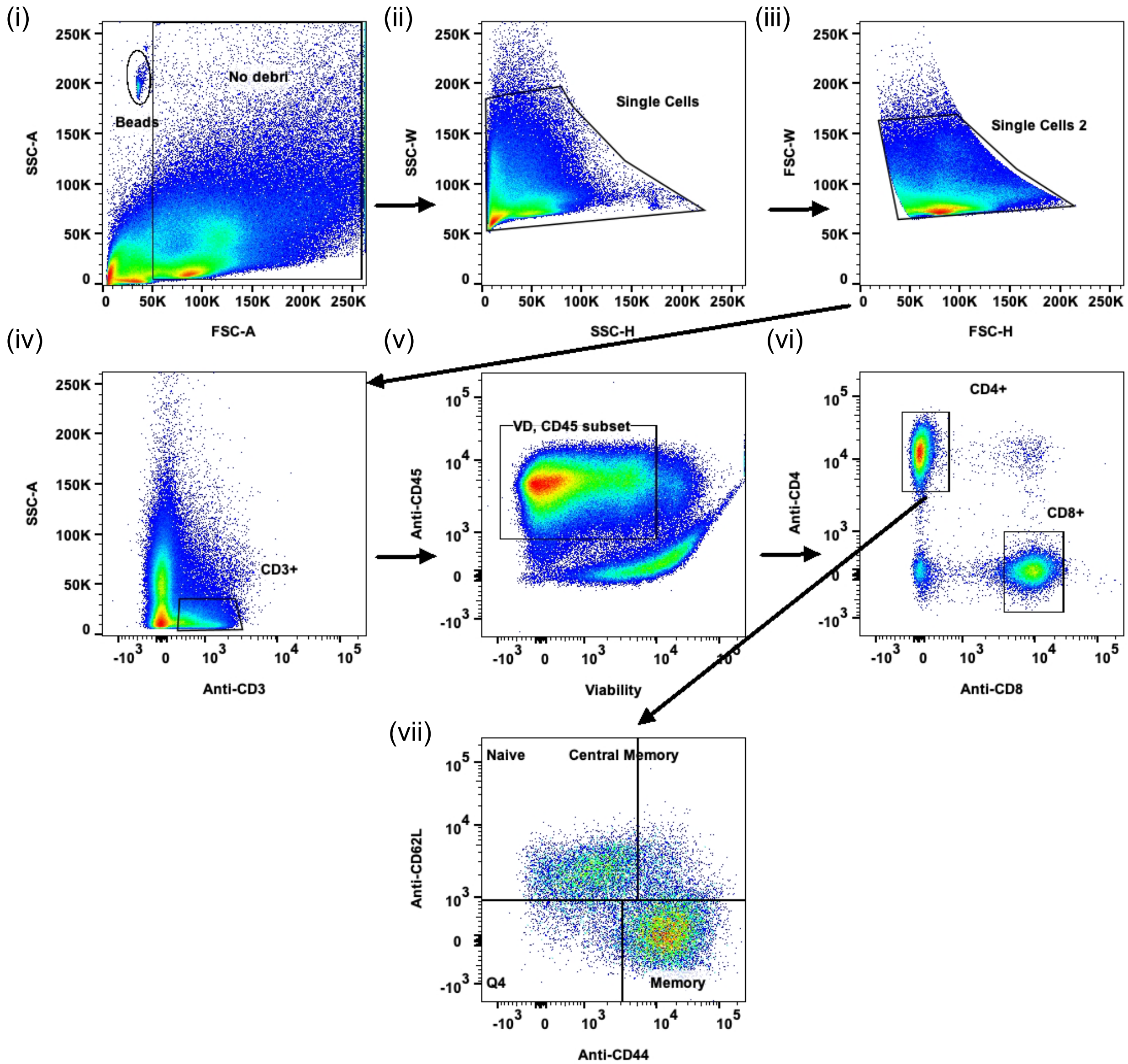

B: Representative gating for T cells in the BM transplant recipients from mixed donors of CD45.2 *Mbl*$^{-/-}$ and CD45.1 wildtype mice.

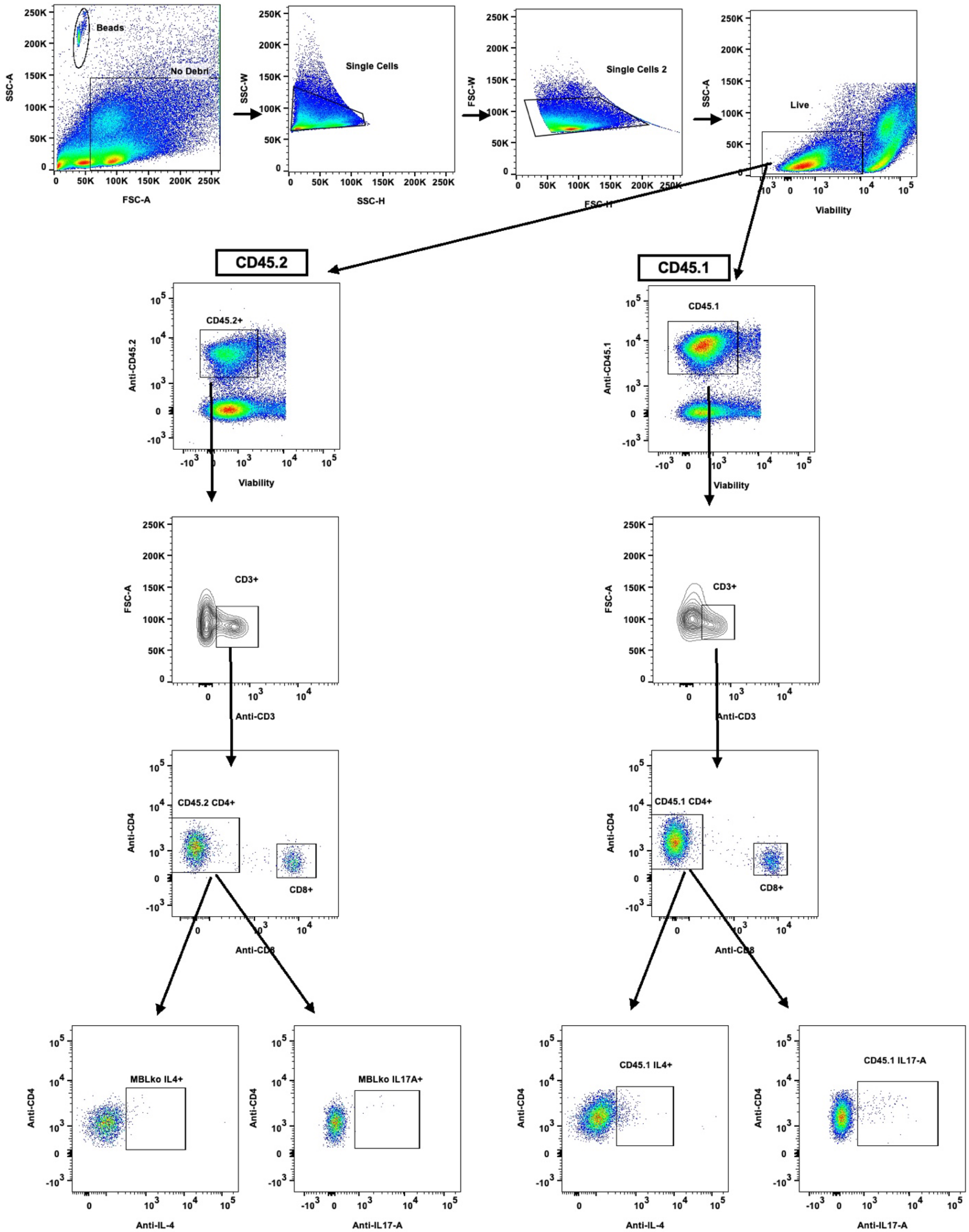

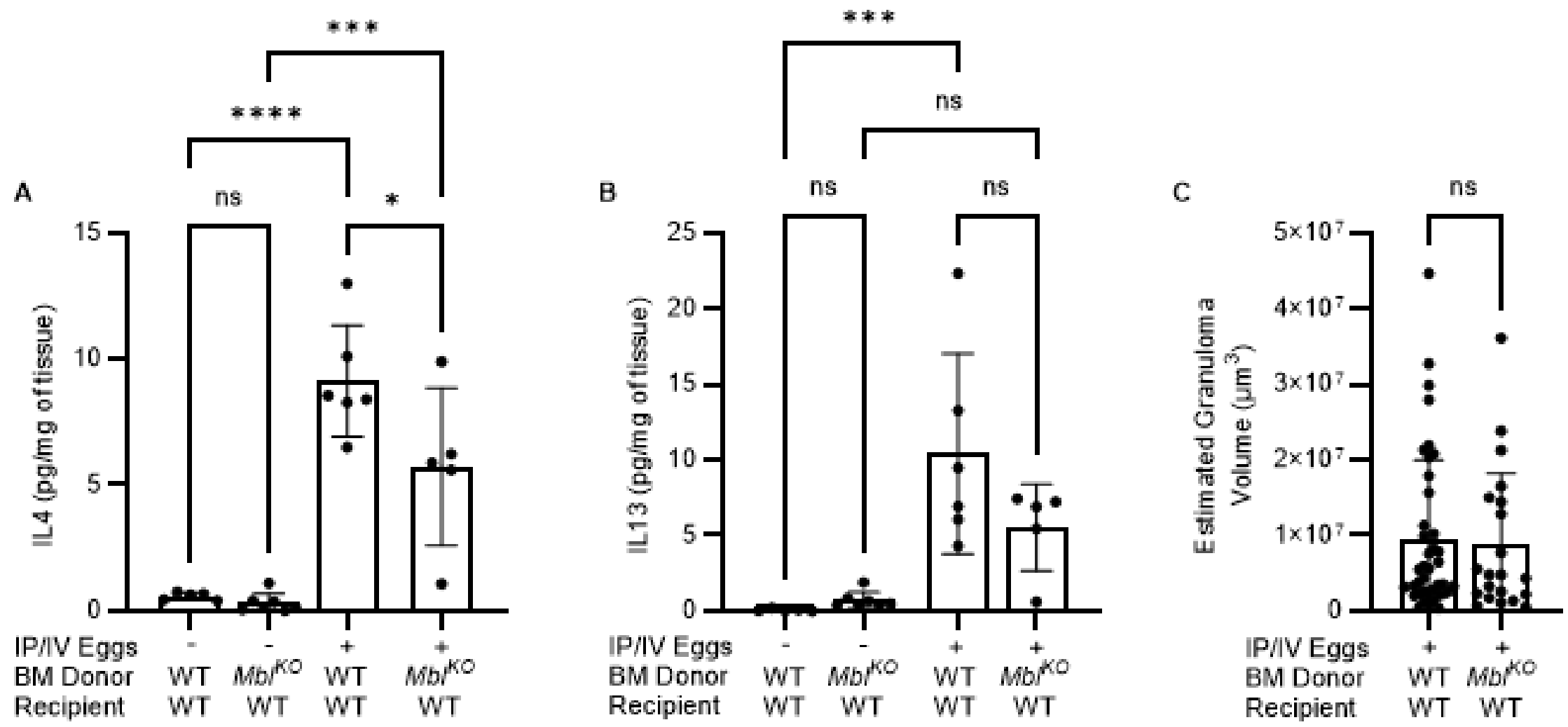

Supplemental Figure 3. Whole lung IL4 and IL13 protein concentrations, and estimated peri-egg granuloma volumes, in either MBL wildtype or knockout mice, unexposed or exposed to IP/IV *Schistosoma* eggs. (A) IL4 and (B) IL13 protein concentrations (N=5-7/group; mean +/- SD plotted; ANOVA with post-hoc Tukey test shown). (C) Estimated peri-egg granuloma volume (N=21-40/group; mean +/- SD plotted; unpaired t-test). Abbreviations: BM: bone marrow; IP: intraperitoneal; IV: intravenous; *MBL*$^{-/-}$: mannose binding lectin 1 and 2 knockout; WT: wildtype. P values: ns: non-significant; *P<0.05, ***P<0.001, ****P<0.0001.

Supplemental Figure 4. In *Schistosoma* IP sensitized and IV challenged IL-5 reporter mice, there are more Th2 cells in the entire lung, but in particular there are more Th2 cells located adjacent to the pulmonary vasculature.

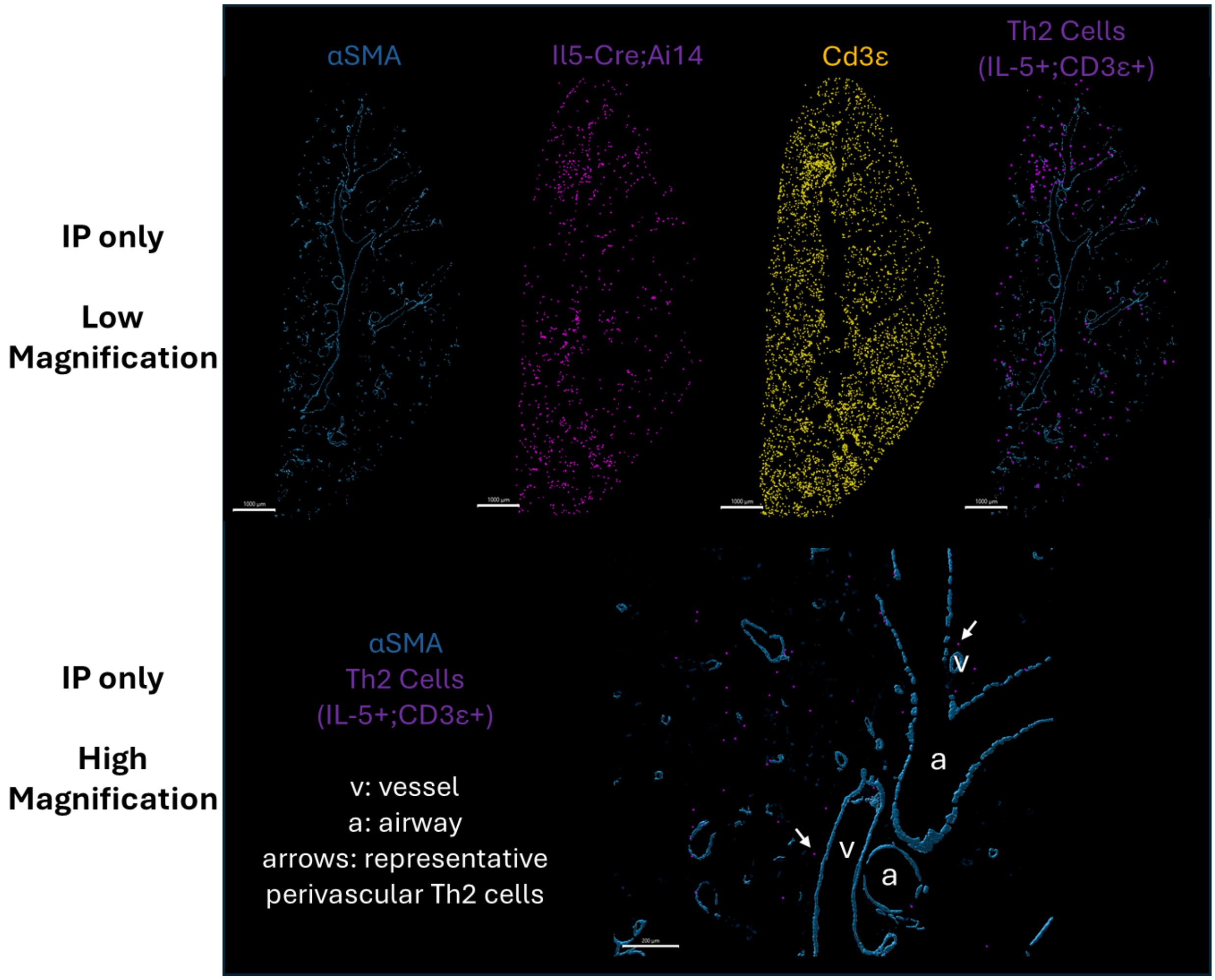

A: *Schistosoma* IP eggs only (does not cause PH). Low and high magnification. Surface projections of alpha-smooth muscle actin (αSMA), *Il5-Cre;Ai14*, and *Cd3ε*. Th2 cells are identified as being double-positive for both IL5 and CD3ε. Low magnification scale bars = 1000 μm; high magnification scale bar = 200 μm; arrows mark representative perivascular Th2 cells.

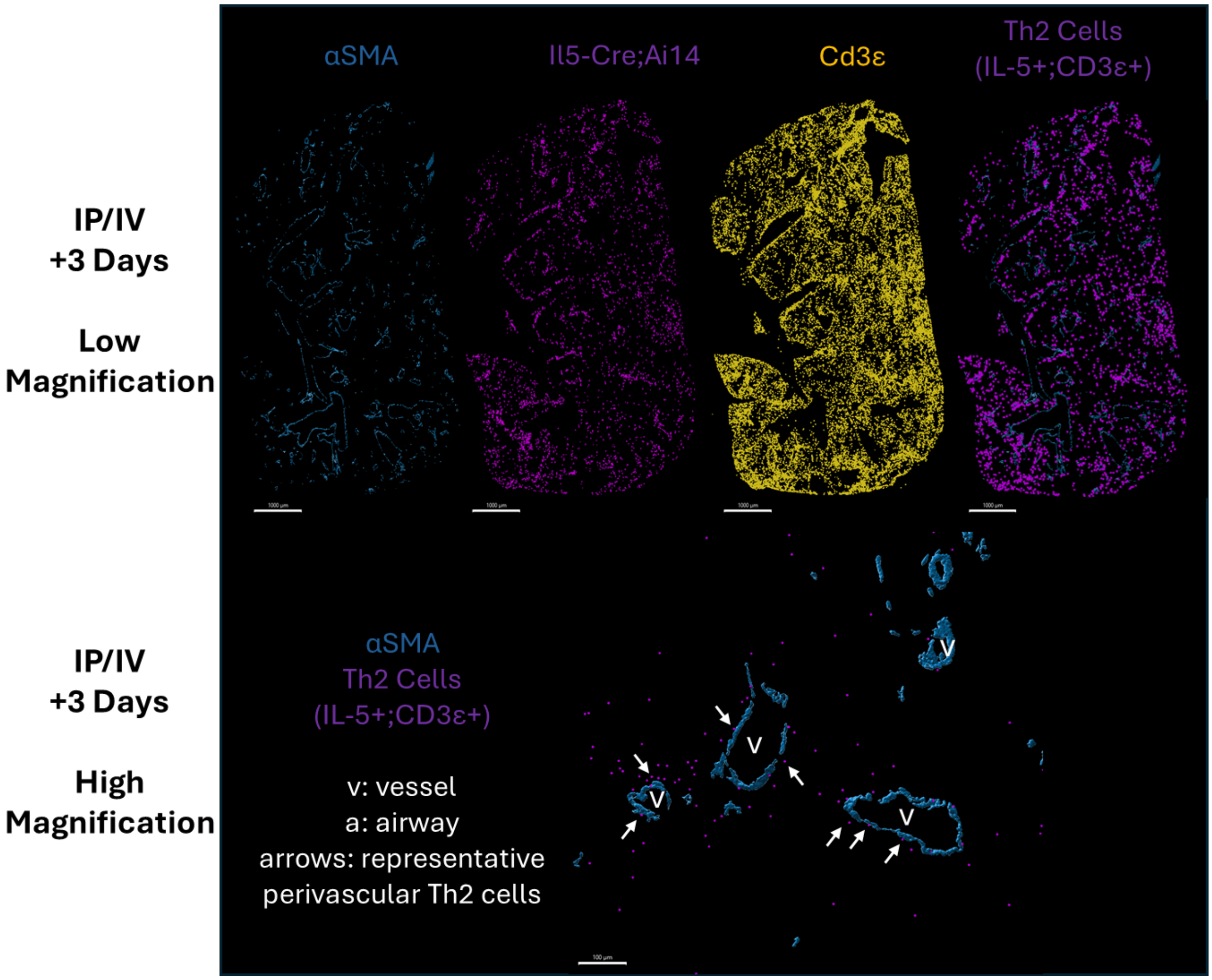

B: *Schistosoma* IP eggs, followed by IV eggs, and sacrificed at +3 days following the IV eggs. Low and high magnification. Surface projections of alpha-smooth muscle actin (αSMA), *Il5-Cre;Ai14*, and *Cd3ε*. Th2 cells are identified as being double-positive for both IL5 and CD3ε. Low magnification scale bars = 1000 μm; high magnification scale bar = 100 μm.

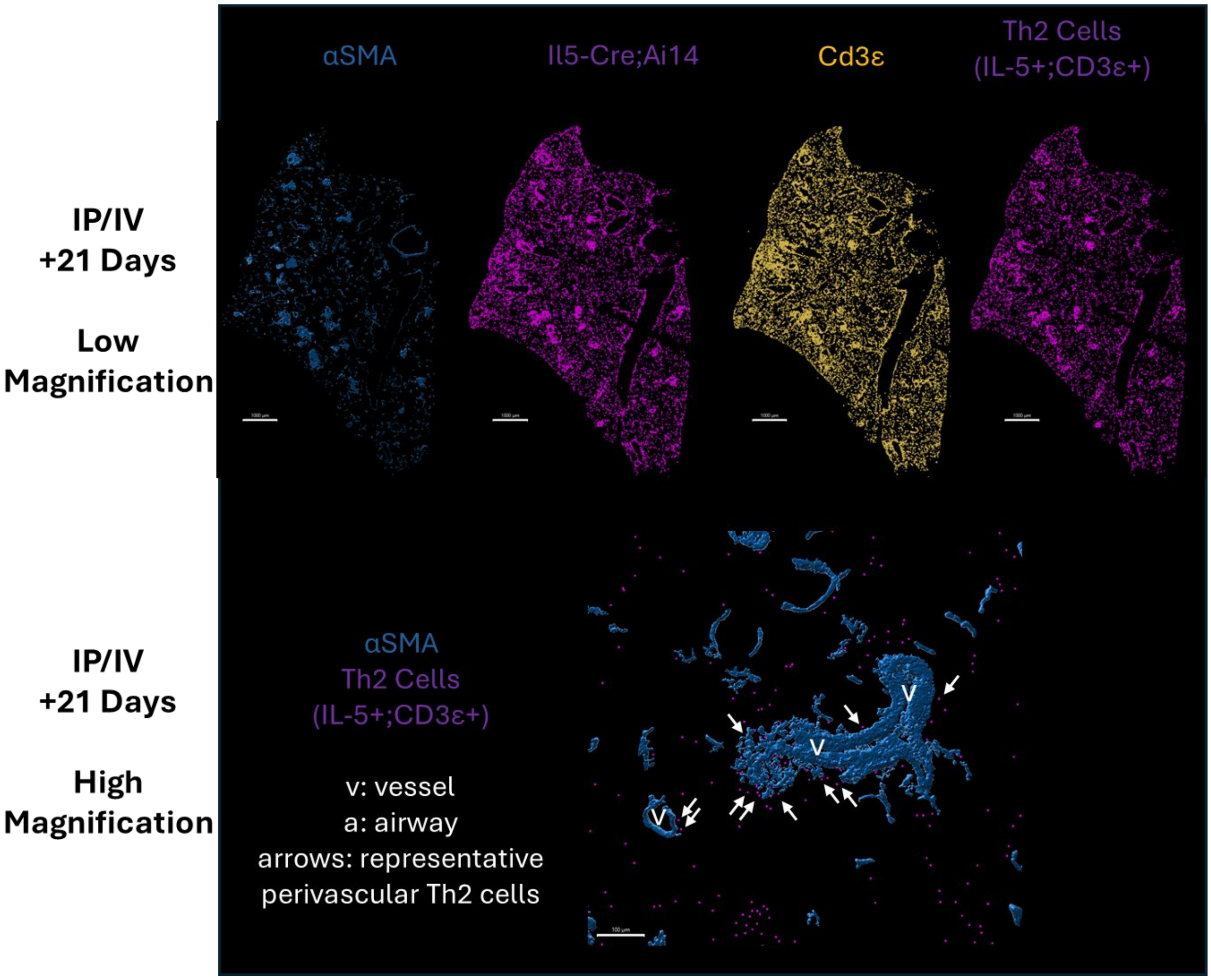

C: *Schistosoma* IP eggs, followed by IV eggs, and sacrificed at +21 days following the IV eggs. Low and high magnification. Surface projections of alpha-smooth muscle actin (αSMA), *Il5-Cre;Ai14*, and *Cd3ε*. Th2 cells are identified as being double-positive for both IL5 and CD3ε. Low magnification scale bars = 1000 µm; high magnification scale bar = 100 µm.

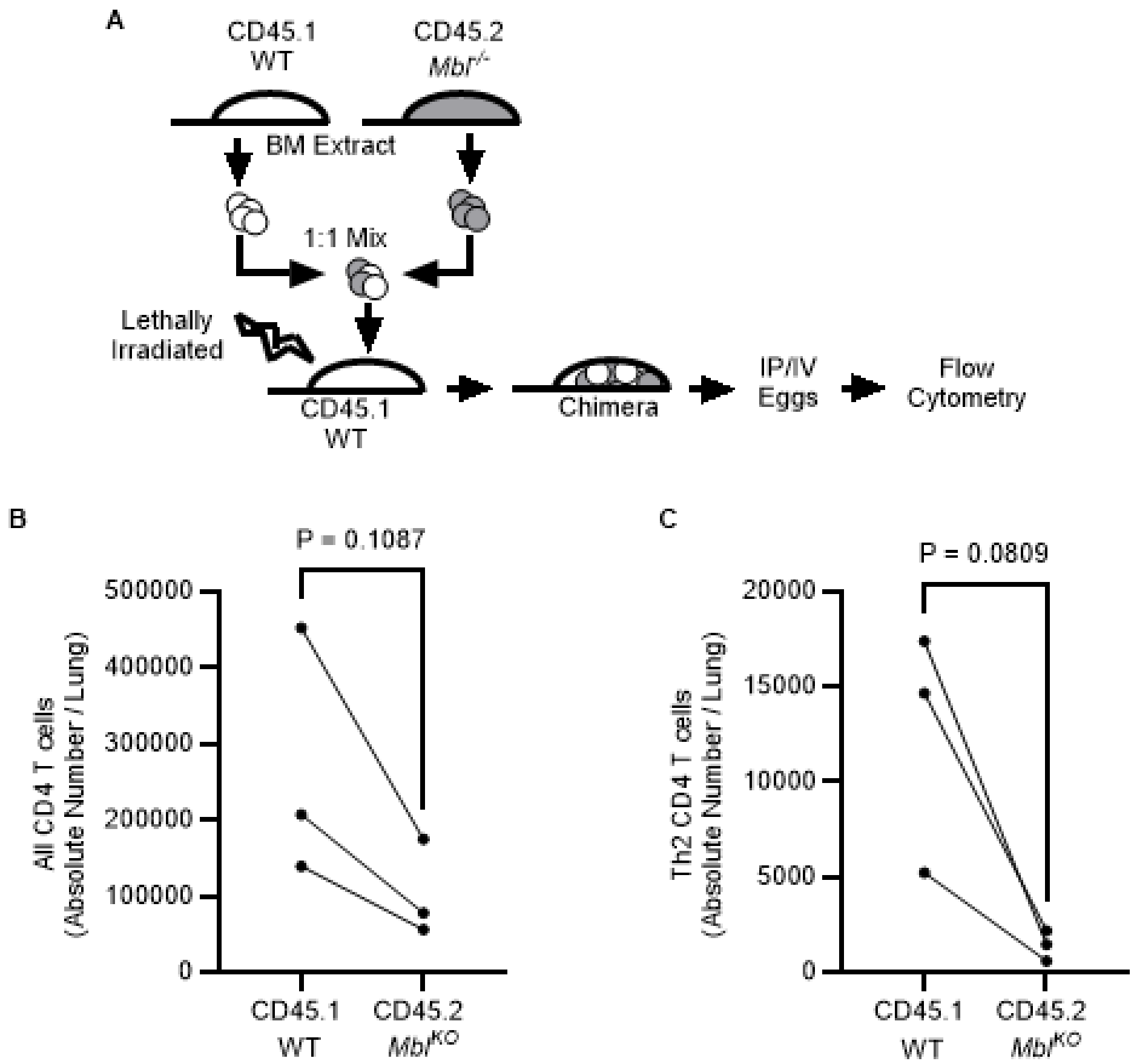

Supplemental Figure 5. MBL expression by CD4 T cells contributes to their Th2 phenotype. (A) Summary of the experimental design to determine the function of MBL expression by T cells *in vivo*. A 1:1 mixture of BM cells from CD45.1 wildtype and CD45.2 Mbl^{KO} mice was transplanted into lethally irradiated CD45.1 wildtype mice. The mice were then subject to *Schistosoma* sensitization and challenge, and flow cytometry performed to identify the number and phenotype of CD45.1 and CD45.2 T cells. Absolute number of (B) CD4 T cells and (C) IL-4$^+$ Th2 CD4 T cells, by CD45.1 or CD45.2 status. N=3/group; lines connect the results from a single animal; P values are paired t-test. Abbreviations: BM: bone marrow; IP: intraperitoneal; IV: intravenous; Mbl^{KO}: deficiency of both *Mbl1* and *Mbl2*; WT: wildtype.

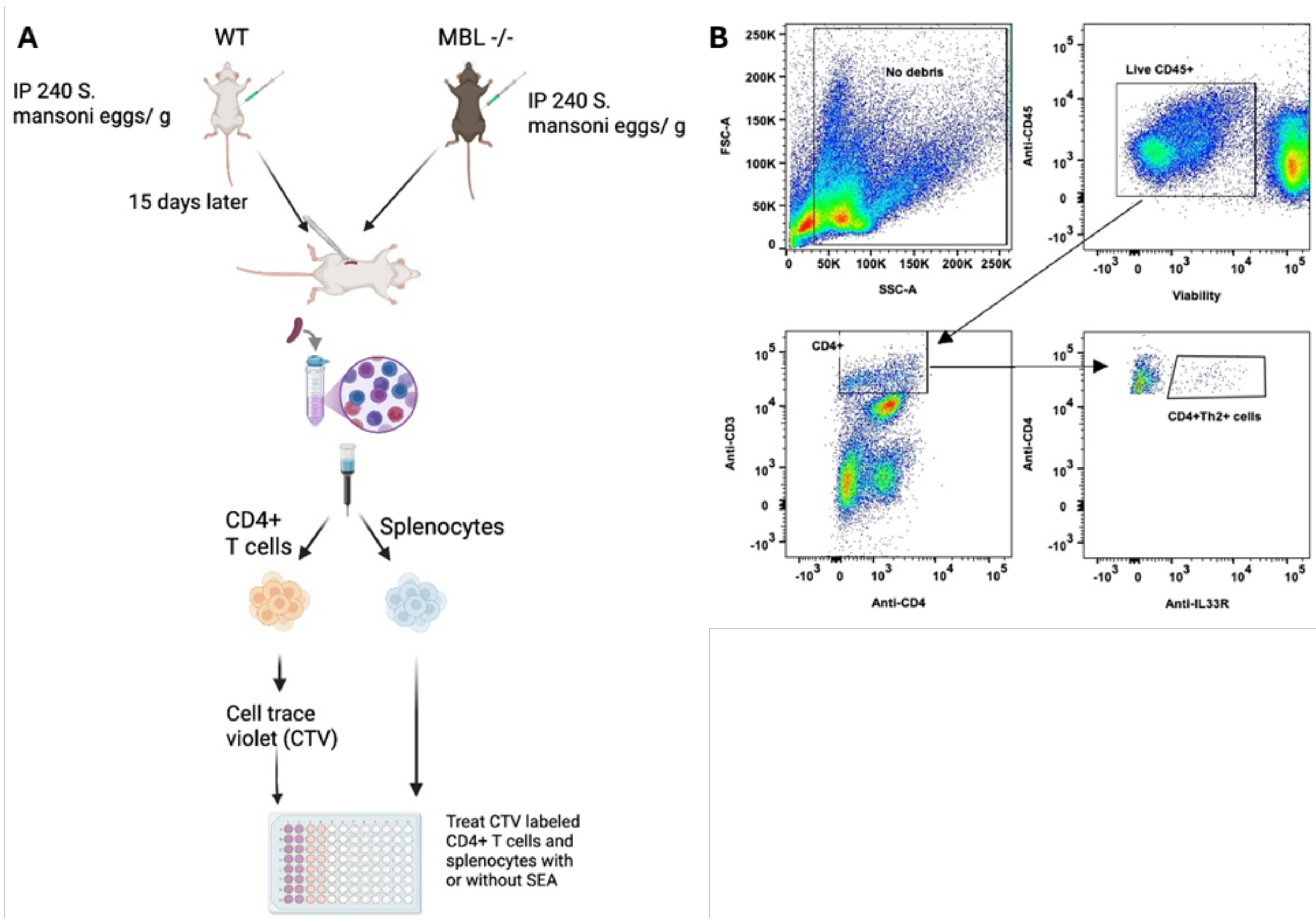

Supplemental Figure 6. (A) Summary of the experimental design to determine the function of MBL expression by T cells in vitro. Bulk splenocytes were harvested from wildtype and *Mbl*$^{-/-}$ mice *Schistosoma*-egg sensitized. CD4 T cells were isolated by magnetic bead separation and labeled with cell trace violet (CTV). The CTV-labeled CD4+T cells, splenocytes, and soluble *S. mansoni* egg antigen (SEA) were then cultured together. (B) The CD4 T cell phenotype was assessed by flow cytometry to isolate CD4 T cells, using the gating strategy shown.

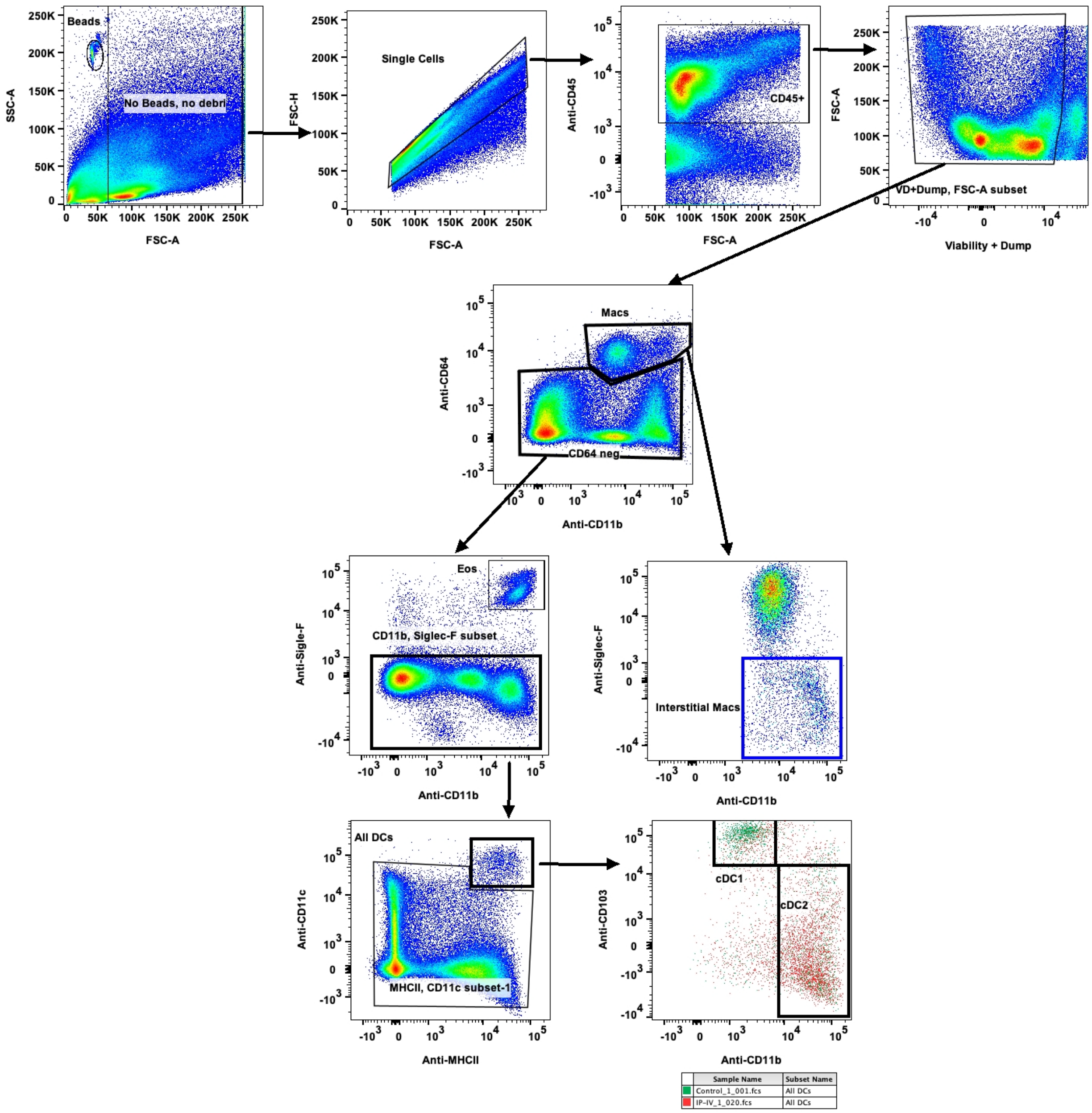

Supplemental Figure 7. Flow cytometry gating strategy to quantify the density of conventional dendritic cells and macrophages in *Mbl*$^{-/-}$ and wildtype chimera mice exposed to *Schistosoma* eggs.

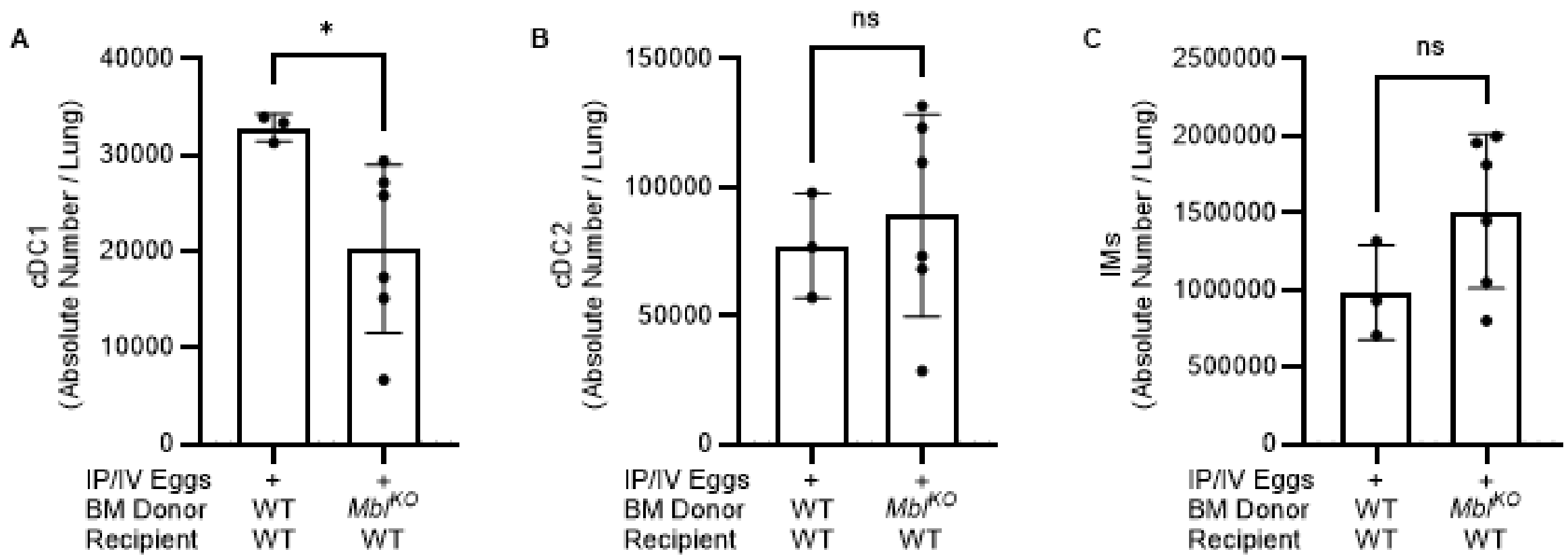

Supplemental Figure 8. Flow cytometry quantification of the density of conventional dendritic cells and macrophages in *Mbl*$^{-/-}$ and wildtype chimera mice exposed to *Schistosoma* eggs. Absolute number of (A) type 1 and (B) type 2 conventional dendritic cells, and (C) interstitial macrophages in the lungs of wildtype chimera or *Mbl*$^{-/-}$ chimera mice (N=3-6/group; mean +/- SD plotted; t-test). Abbreviations: BM: bone marrow; cDC1: type 1 conventional dendritic cell; cDC2: type 2 conventional dendritic cells; IM: interstitial macrophage; IP: intraperitoneal; IV: intravenous; *Mbl*$^{-/-}$: mannose binding lectin 1 and 2 knockout; WT: wildtype. P values: ns: non-significant; *P<0.05.
