# Supplementary material for: Role of CD4+ T cell mannose binding lectin in Schistosoma-induced pulmonary hypertension
Source: JCI Insight. 2026 Jul 28;11(17):e202919. doi: 10.1172/jci.insight.202919 (PMC13564070; doi:10.1172/jci.insight.202919)
Supplement: Supplementary file 2 [file jciinsight-11-202919-s194.pdf]

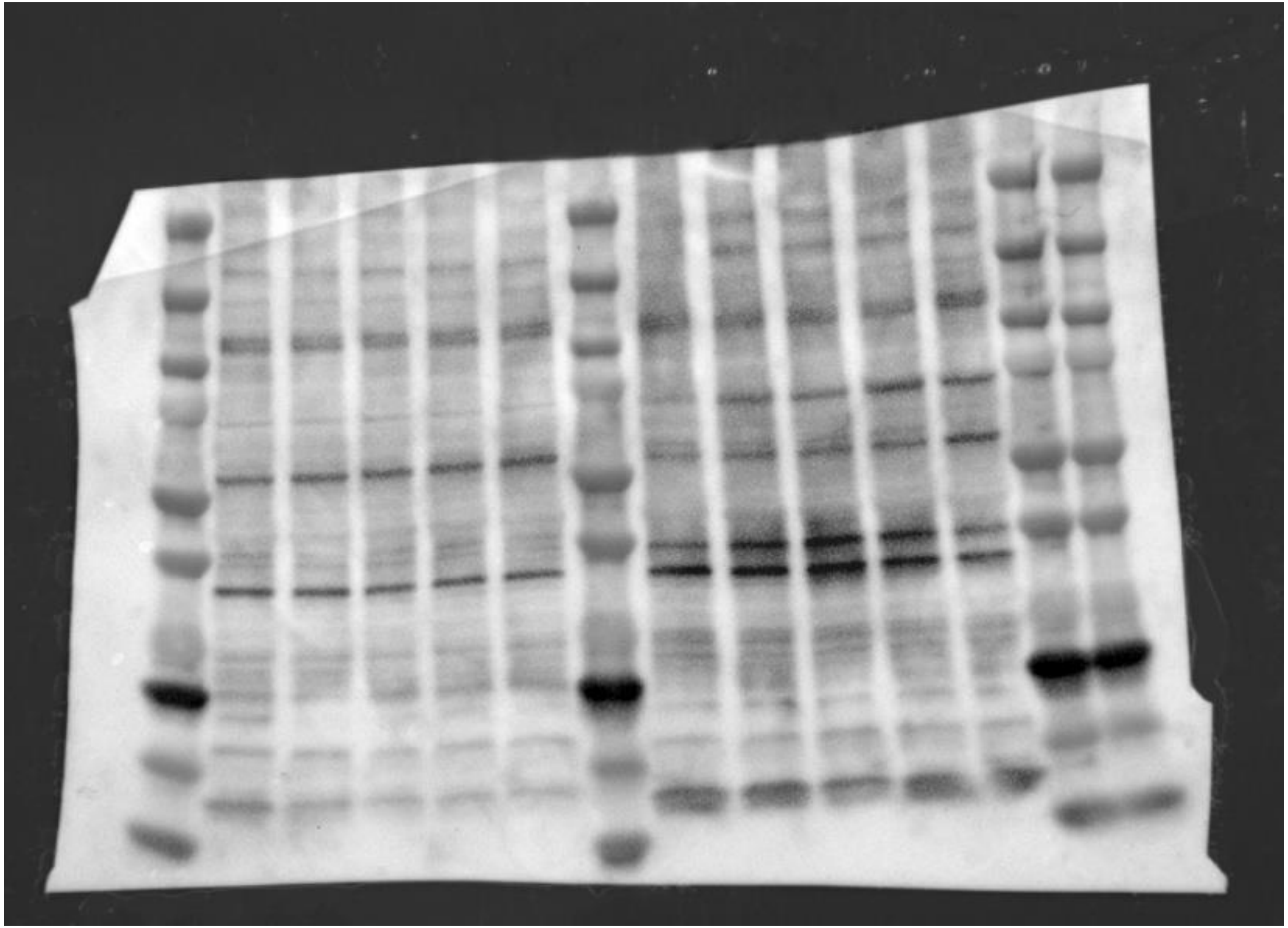

Full unedited gel for Supplemental Figure 1A: upper left western blot (WT chimera, anti-C3)

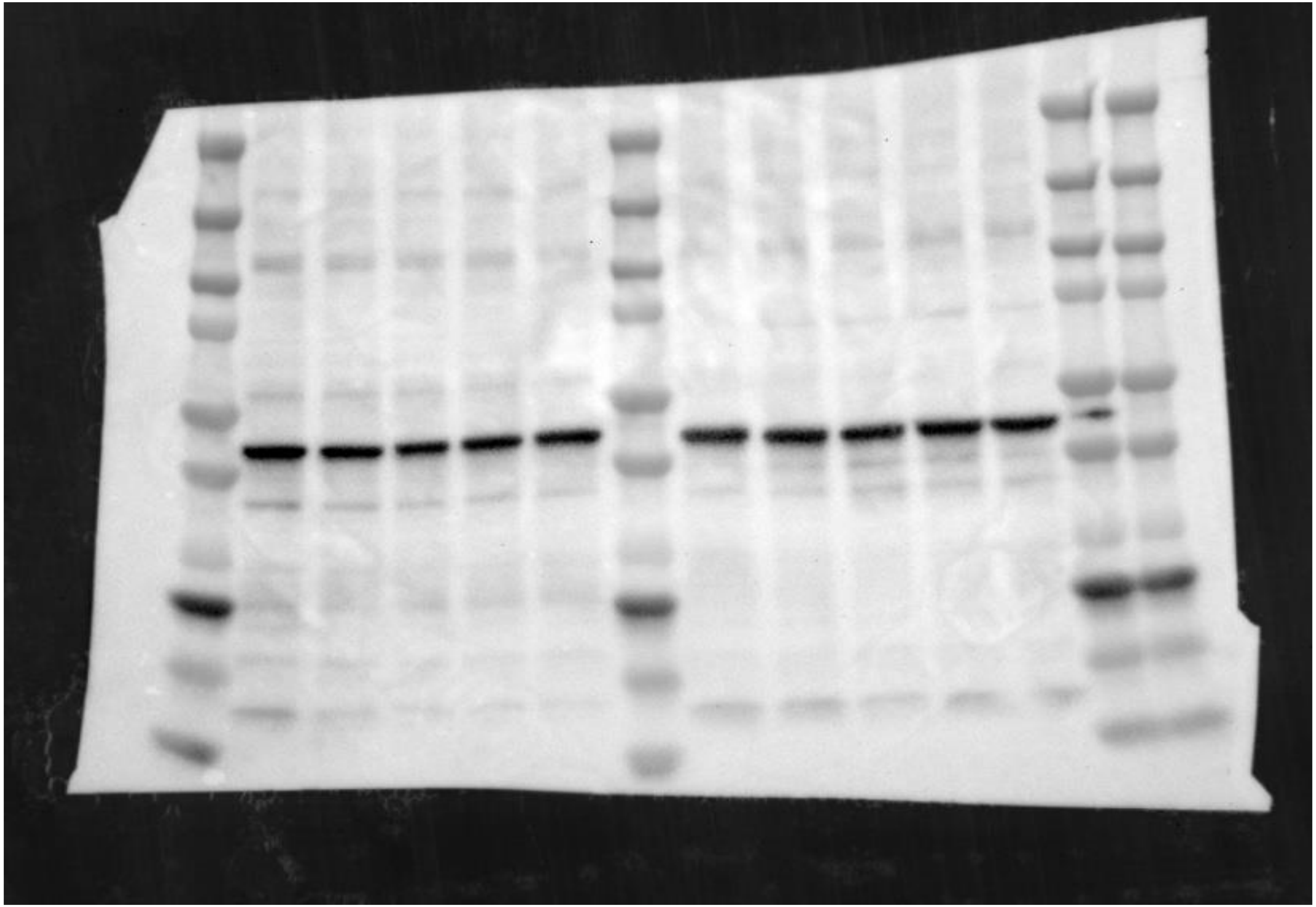

Full unedited gel for Supplemental Figure 1A: lower left western blot (WT chimera, anti-beta actin)

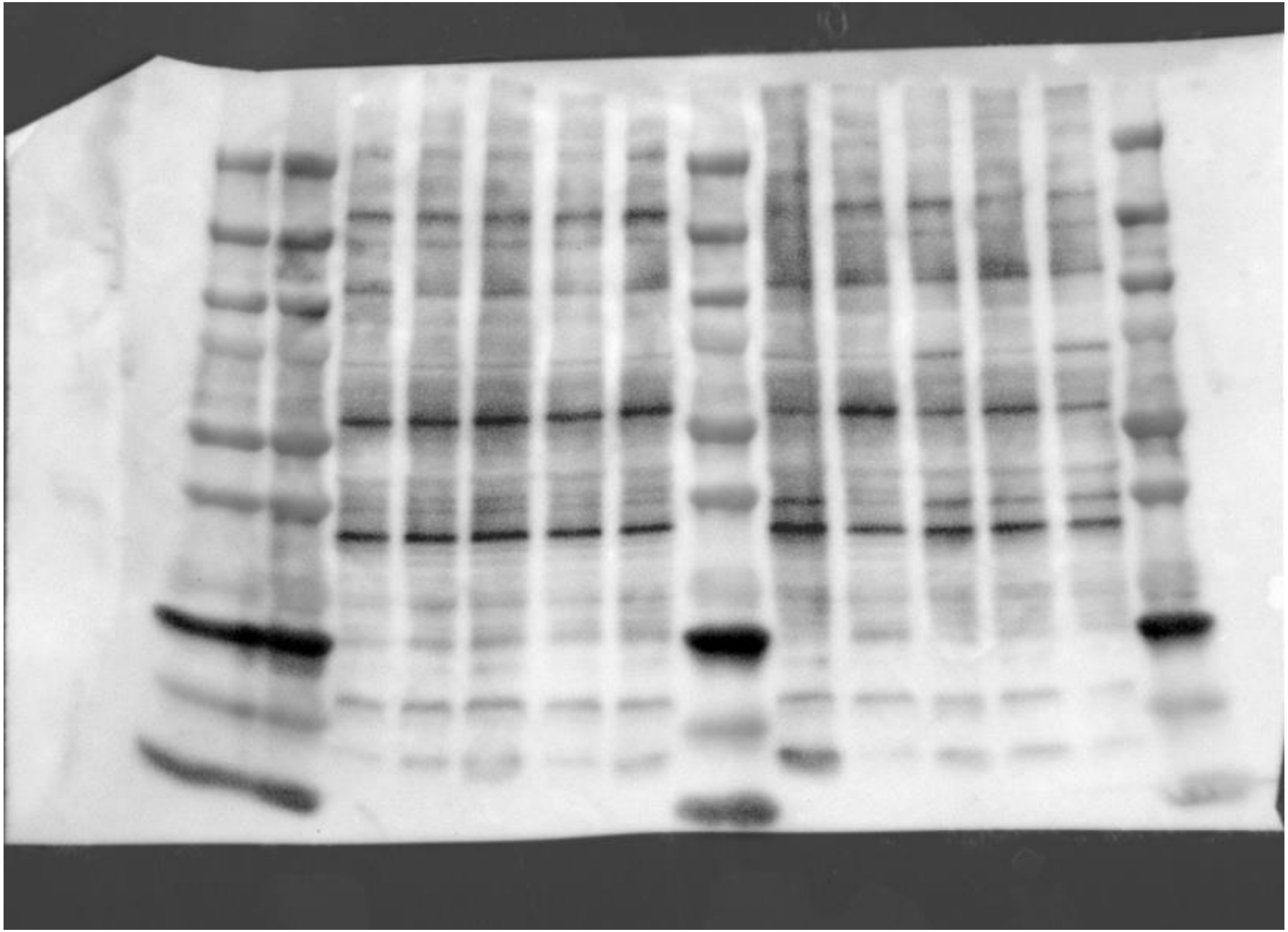

Full unedited gel for Supplemental Figure 1A: upper right western blot (MBL-KO chimera, anti-C3)

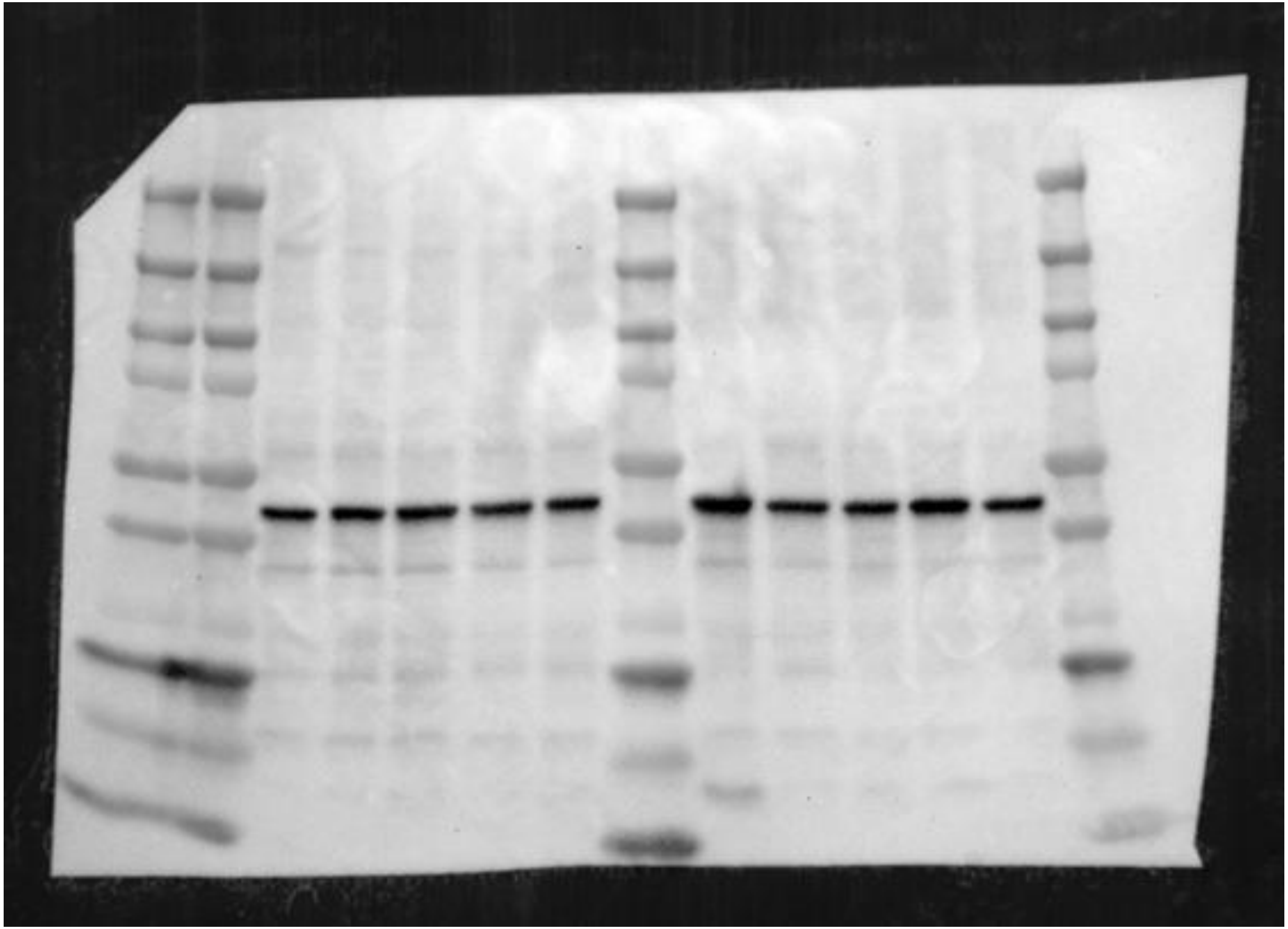

Full unedited gel for Supplemental Figure 1A: lower right western blot (MBL-KO chimera, anti-beta actin)
